# Stereoselective synthesis of the C79–C97 fragment of symbiodinolide

**DOI:** 10.3762/bjoc.9.228

**Published:** 2013-09-25

**Authors:** Hiroyoshi Takamura, Takayuki Fujiwara, Isao Kadota, Daisuke Uemura

**Affiliations:** 1 Department of Chemistry, Graduate School of Natural Science and Technology, Okayama University, 3-1-1 Tsushimanaka, Kita-ku, Okayama 700-8530, Japan; 2 Department of Chemistry, Faculty of Science, Kanagawa University, 2946 Tsuchiya, Hiratsuka 259-1293, Japan

**Keywords:** Julia–Kocienski olefination, polyol marine natural product, Sharpless asymmetric dihydroxylation, spiroacetalization, symbiodinolide

## Abstract

Symbiodinolide is a polyol marine natural product with a molecular weight of 2860. Herein, a streamlined synthesis of the C79–C97 fragment of symbiodinolide is described. In the synthetic route, a spiroacetalization, a Julia–Kocienski olefination, and a Sharpless asymmetric dihydroxylation were utilized as the key transformations.

## Findings

**Figure 1:**
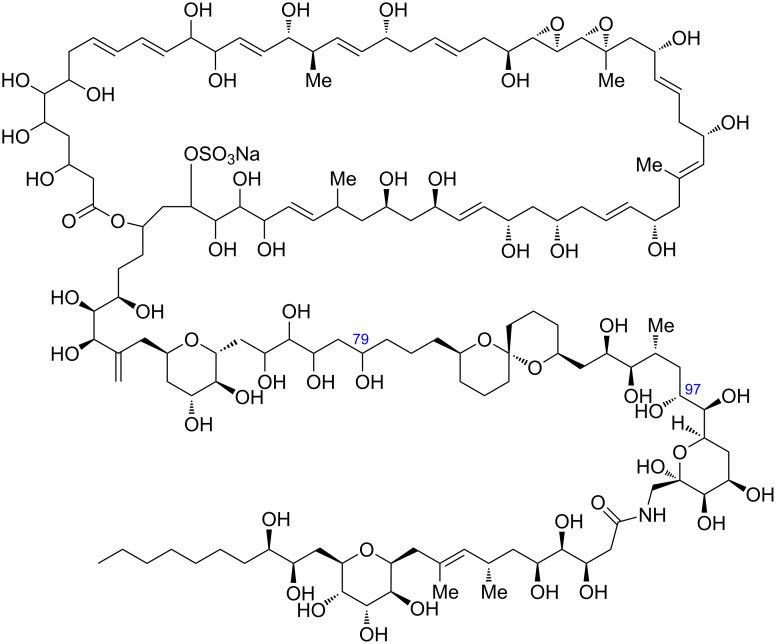
Structure of symbiodinolide (**1**).

A 62-membered polyol marine natural product, symbiodinolide (**1**, Figure 1), was isolated from the 80% aqueous ethanol extract of the cultured symbiotic dinoflagellate *Symbiodinium* sp. in 2007 [1]. Symbiodinolide shows voltage-dependent N-type Ca^2+^ channel-opening activity at 7 nmol/L and COX-1 inhibitory effect at 2 μmol/L (65% inhibition). Furthermore, **1** ruptures the tissue surface of the acoel flatworm *Amphiscolops* sp. at 2.5 μmol/L. The entire planar structure of **1** was established by the detailed 2D NMR spectroscopic analysis. However, the complete stereostructural determination of **1** with its 61 chirality centres and a molar mass of 2860 has been an unsolved issue. Therefore, in order to complete the configurational elucidation of **1**, we are now investigating its chemical degradation [1–3] and chemical synthesis of the fragments [4–11].

**Scheme 1:**
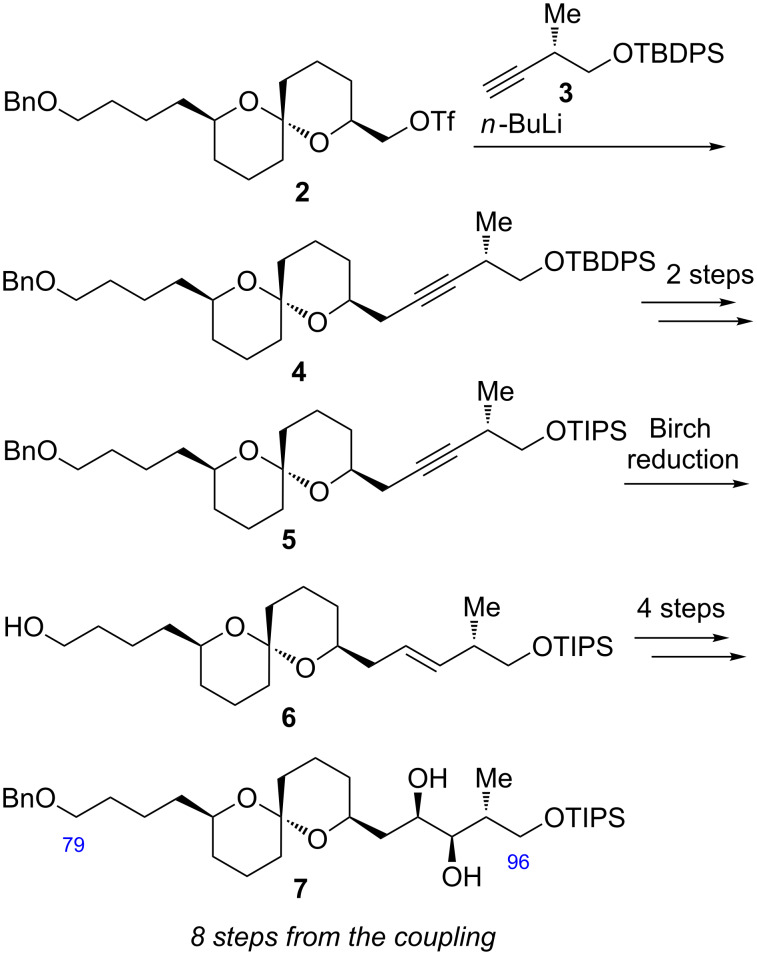
Our previous synthesis of the C79–C96 fragment **7**.

Previously, we reported the stereoselective synthesis of the spiroacetal C79–C96 fragment [4], which is summarized in Scheme 1. Triflate **2** was reacted with the lithium acetylide prepared from alkyne **3** to give the desired coupling product **4**. The TBDPS ether **4** was transformed to TIPS ether **5** because of the lability of the TBDPS protecting group under the following Birch conditions. The alkyne **5** was subjected to the Birch reduction to afford the *trans*-alkene **6**, wherein the benzyl moiety was deprotected. The alkene **6** was derivatized to the spiroacetal C79–C96 fragment **7** in four steps including the benzyl protection and Sharpless asymmetric dihydroxylation (AD). Although the desired spiroacetal fragment **7** was synthesized stereoselectively, the transformation starting with the coupling between **2** and **3** to the final product **7** needed eight steps. Therefore, we decided to examine the more efficient synthesis of the spiroacetal fragment. Herein, we report the improved synthesis of the spiroacetal fragment by utilizing Julia–Kocienski olefination as the coupling reaction.

**Scheme 2:**
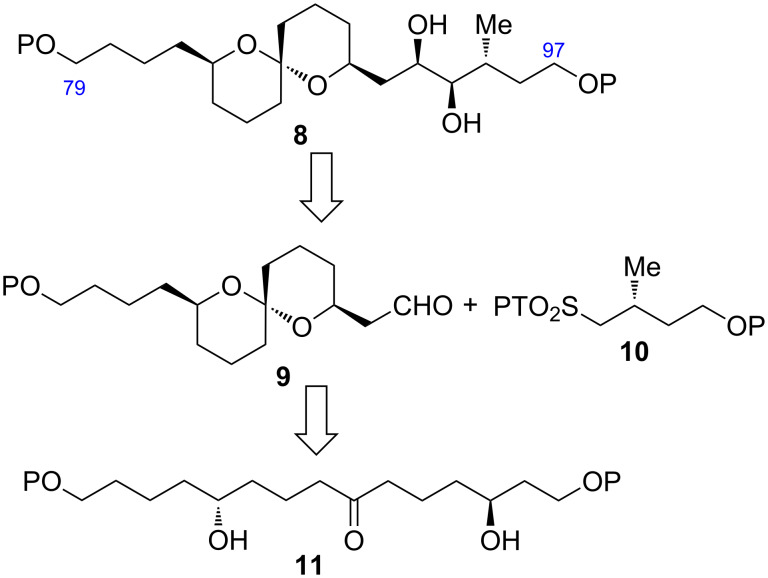
Retrosynthetic analysis of the C79–C97 fragment **8**.

The new retrosynthetic analysis of the C79–C97 fragment **8** is described in Scheme 2. We envisaged that the diol **8** could be synthesized by the Julia–Kocienski olefination [12–14] between aldehyde **9** and 1-phenyl-1*H*-tetrazol-5-yl (PT)-sulfone **10** and subsequent Sharpless AD [15], wherein the target molecule **8** could be prepared in two steps from the coupling. The carbon framework of **9** could be constructed through the stereoselective spiroacetalization of dihydroxyketone **11**.

**Scheme 3:**
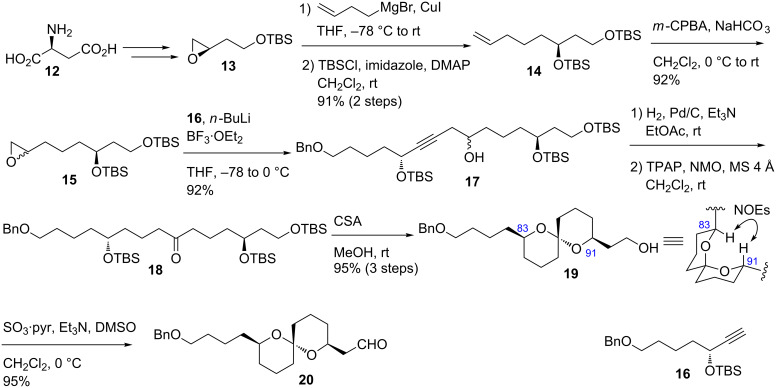
Synthesis of aldehyde **20**.

First, we commenced the stereocontrolled synthesis of aldehyde **20** (Scheme 3). Treatment of epoxide **13**, which was prepared from L-aspartic acid (**12**) by the known procedure [8], with 3-butenylmagnesium bromide/CuI [16] provided the corresponding secondary alcohol. Protection of the alcohol with TBSCl afforded TBS ether **14** in 91% yield in two steps. Alkene **14** was reacted with *m*-CPBA to produce epoxide **15** as a 1:1 diastereomeric mixture. Epoxide **15** was coupled with alkyne **16** [4] in the presence of *n*-BuLi/BF_3_∙OEt_2_ [17] to give the desired product **17** in 92% yield from **15**. Hydrogenation of the alkyne moiety of **17** followed by TPAP oxidation [18] yielded ketone **18**. Removal of the three TBS protecting groups and subsequent stereoselective spiroacetalization were performed in one-pot with CSA in MeOH to provide spiroacetal **19** as a single stereoisomer [19–20]. The stereochemistry of **19** was elucidated by the observed NOE correlations between H-83 and H-91 as indicated by an arrow. The plausible rationale for the stereoselective formation of **19** is the thermodynamic stability due to the double anomeric effect. Oxidation of the alcohol **19** with SO_3_∙pyr/Et_3_N/DMSO [21] afforded aldehyde **20**.

**Scheme 4:**
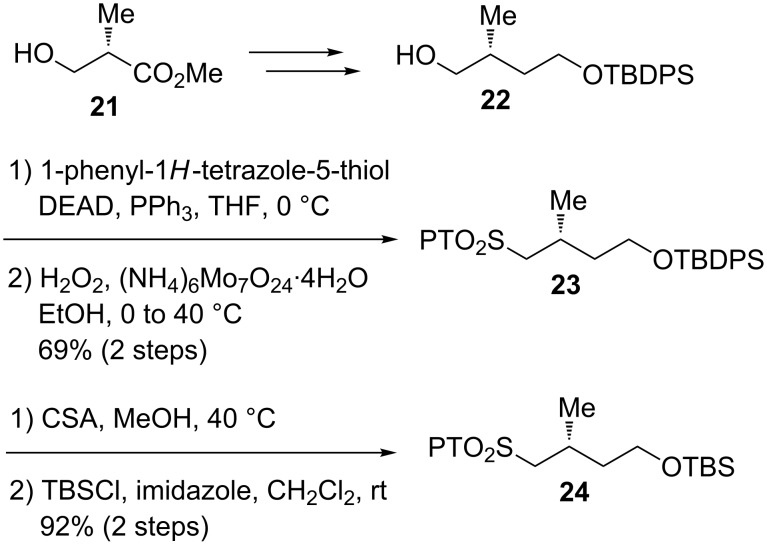
Synthesis of PT-sulfones **23** and **24**.

Next, we carried out the synthesis of PT-sulfones **23** and **24** which were the coupling partners of the aldehyde **20** (Scheme 4). The synthesis started from commercially available methyl (*S*)-3-hydroxy-2-methylpropanoate (**21**), which was converted to alcohol **22** by the known method [22]. Alcohol **22** was treated with 1-phenyl-1*H*-tetrazole-5-thiol/DEAD/PPh_3_ to furnish the corresponding PT-sulfide, which was oxidized with H_2_O_2_/Mo(VI) [23] to yield PT-sulfone **23**. The TBDPS protecting group of **23** was transformed to the TBS group in two steps to provide PT-sulfone **24**.

**Scheme 5:**
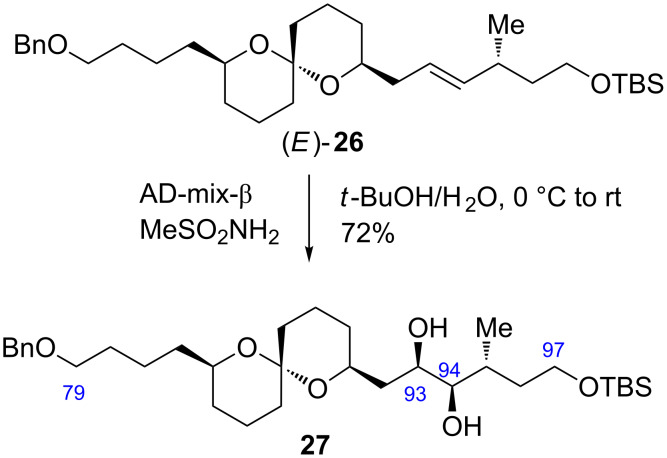
Synthesis of the C79–C97 fragment **27**.

With the coupling precursors aldehyde **20** and PT-sulfones **23** and **24** in hand, we next examined the Julia–Kocienski olefination [12–14] of these compounds (Table 1). Deprotonation of the PT-sulfone **23** with KHMDS, followed by addition of the aldehyde **20**, gave rise to the desired coupling product (*E*)-**25** along with (*Z*)-**25** in 27% combined yield at a 3.5:1 diastereomeric ratio (Table 1, entry 1). When NaHMDS was used as the base, the chemical yield was improved to 77%, however, the *E*/*Z* ratio was decreased to 1.3:1 (Table 1, entry 2). When LDA was used as the base, the chemical yield and diastereomeric ratio were increased to 98% and 2.6:1, respectively (Table 1, entry 3). Reaction of PT-sulfone **24** using LDA gave the coupling products (*E*)- and (*Z*)-**26** in 86% yield, wherein the diastereomeric ratio was increased to 5.0:1 (Table 1, entry 4). The configurations of the coupling products were elucidated by their coupling constants between H-93 and H-94 (15.3 Hz in (*E*)-**25** and (*E*)-**26**, 10.7 Hz in (*Z*)-**25** and (*Z*)-**26**). Finally, the desired alkene (*E*)-**26** was subjected to the Sharpless AD [15] with AD-mix-β to furnish the C79–C97 fragment **27** in 72% yield as a single diastereomer (Scheme 5). The configuration of the resulting two vicinal hydroxy groups at C93 and C94 of **27** were unambiguously confirmed by the modified Mosher method, respectively (see Supporting Information File 1).

**Table 1:** Julia–Kocienski olefination between aldehyde **20** and PT-sulfones **23** and **24**.

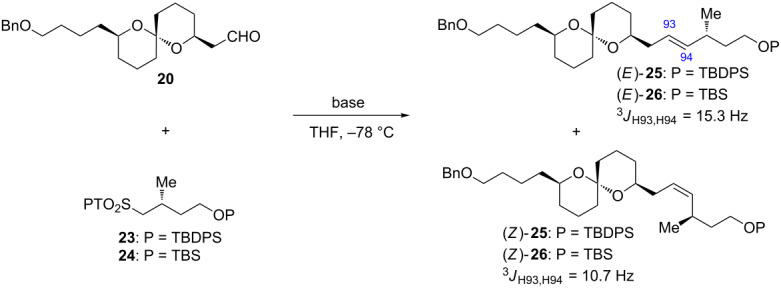				

Entry	PT-Sulfone	Base	Yield (%)^a^	Ratio (*E*:*Z*)^b^

1	**23**	KHMDS	27	3.5:1
2	**23**	NaHMDS	77	1.3:1
3	**23**	LDA	98	2.6:1
4	**24**	LDA	86	5.0:1

^a^Isolated yield from **20**. ^b^Determined by ^1^H NMR spectroscopy.

In conclusion, we have achieved the stereoselective synthesis of the C79–C97 fragment. The synthetic route has featured a stereoselective spiroacetalization, a Julia–Kocienski olefination, and a Sharpless asymmetric dihydroxylation. This synthesis of the spiroacetal fragment, wherein the two-step sequence was conducted and the overall yield was 52% from the coupling, has been improved over the previous synthesis wherein the eight-step transformation was needed and the overall yield was 31% from the coupling. Further synthetic effort of symbiodinolide toward the complete structural elucidation is currently underway and will be reported in due course.

## Supporting Information

File 1 Experimental procedures, spectroscopic data, and NMR spectra of all new compounds.

## References

[1] Kita M, Ohishi N, Konishi K, Kondo M, Koyama T, Kitamura M, Yamada K, Uemura D (2007). Tetrahedron.

[2] Han C, Uemura D (2008). Tetrahedron Lett.

[3] Han C, Yamano Y, Kita M, Takamura H, Uemura D (2009). Tetrahedron Lett.

[4] Takamura H, Ando J, Abe T, Murata T, Kadota I, Uemura D (2008). Tetrahedron Lett.

[5] Murata T, Sano M, Takamura H, Kadota I, Uemura D (2009). J Org Chem.

[6] Takamura H, Murata T, Asai T, Kadota I, Uemura D (2009). J Org Chem.

[7] Takamura H, Kadonaga Y, Yamano Y, Han C, Aoyama Y, Kadota I, Uemura D (2009). Tetrahedron Lett.

[8] Takamura H, Kadonaga Y, Yamano Y, Han C, Kadota I, Uemura D (2009). Tetrahedron.

[9] Takamura H, Kadonaga Y, Kadota I, Uemura D (2010). Tetrahedron Lett.

[10] Takamura H, Kadonaga Y, Kadota I, Uemura D (2010). Tetrahedron.

[11] Takamura H, Tsuda K, Kawakubo Y, Kadota I, Uemura D (2012). Tetrahedron Lett.

[12] Blakemore P R, Cole W J, Kocieński P J, Morley A (1998). Synlett.

[13] Aïssa C (2009). Eur J Org Chem.

[14] Blakemore P R (2002). J Chem Soc, Perkin Trans 1.

[15] Kolb H C, VanNieuwenhze M S, Sharpless K B (1994). Chem Rev.

[16] Paterson I, Anderson E A, Dalby S M, Lim J H, Maltas P, Loiseleur O, Genovino J, Moessner C (2012). Org Biomol Chem.

[17] Yamaguchi M, Hirao I (1983). Tetrahedron Lett.

[18] Ley S V, Norman J, Griffith W P, Marsden S P (1994). Synthesis.

[19] Perron F, Albizati K F (1989). Chem Rev.

[20] Aho J E, Pihko P M, Rissa T K (2005). Chem Rev.

[21] Parikh J R, Doering W v E (1967). J Am Chem Soc.

[22] Kurosawa S, Mori K (2000). Eur J Org Chem.

[23] Schultz H S, Freyermuth H B, Buc S R (1963). J Org Chem.

