# Supplementary material for: Stereoselective synthesis of the C79–C97 fragment of symbiodinolide
Source: Beilstein J Org Chem. 2013 Sep 25;9:1931–5. doi: 10.3762/bjoc.9.228 (PMC3817580; doi:10.3762/bjoc.9.228)
Supplement: File 1 — Experimental procedures, spectroscopic data, and NMR spectra of all new compounds. [file Beilstein_J_Org_Chem-09-1931-s001.pdf]

Supporting Information

for

Stereoselective synthesis of the C79–C97 fragment of symbiodinolide

Hiroyoshi Takamura*[1], Takayuki Fujiwara[1], Isao Kadota*[1] and Daisuke Uemura[2]

Address: [1]Department of Chemistry, Graduate School of Natural Science and Technology, Okayama University, 3-1-1 Tsushimanaka, Kita-ku, Okayama 700-8530, Japan and [2]Department of Chemistry, Faculty of Science, Kanagawa University, 2946 Tsuchiya, Hiratsuka 259-1293, Japan

Email: Hiroyoshi Takamura - takamura@cc.okayama-u.ac.jp; Isao Kadota - kadota-i@cc.okayama-u.ac.jp

* Corresponding author

Experimental procedures, spectroscopic data, and NMR spectra of all new compounds

Table of contents

General methods	S2
Experimental procedures and spectroscopic data of **14**, **15**, **17**, **19**, and **20**	S2–S5
Experimental procedures and spectroscopic data of **23** and **24**	S5–S6
Experimental procedures and spectroscopic data of (*E*)-**26** and **27**	S6–S7
Stereochemical confirmation at the C93 and C94 positions of **27**	S7
Scheme S1: Derivatization of **27** for the stereochemical confirmation.	S7
Figure S1: Chemical shift differences ($\Delta\delta_{S-R}$) of **S1**/**S2** (a) and **S4**/**S5** (b).	S8
Experimental procedures and spectroscopic data of **S1**, **S2**, **S3**, **S4**, and **S5**	S8–S10
NMR spectra of **14**, **15**, **17**, **19**, and **20**	S11–S20
NMR spectra of **23** and **24**	S21–S24
NMR spectra of (*E*)-**26** and **27**	S25–S28
NMR spectra of **S1**, **S2**, **S3**, **S4**, and **S5**	S29–S36

General methods. Reagents were used as received from commercial suppliers unless otherwise indicated. All reactions were carried out under an atmosphere of argon. Reaction solvents were purchased as dehydrated solvents and stored with active molecular sieves 4 Å under argon prior to use for reactions. All solvents for work-up procedures were used as received. Analytical thin-layer chromatography (TLC) was performed with aluminium TLC plates (Merck TLC silica gel $60F_{254}$). Column chromatography was performed with Fuji Silysia silica gel BW-300 or Kanto Chemical silica gel 60N. Optical rotations were recorded on a JASCO DIP-1000. IR spectra were recorded on a JASCO FT/IR-460 plus. ^{1}H and ^{13}C NMR spectra were recorded on JEOL JNM-AL400. Chemical shifts are reported in ppm with reference to the internal residual solvent (^{1}H NMR, $CHCl_3$ 7.26 ppm, C_6H_6 7.15 ppm; ^{13}C NMR, $CDCl_3$ 77.0 ppm, C_6D_6 128.0 ppm). The following abbreviations are used to designate the multiplicities: s = singlet, d = doublet, t = triplet, q = quartet, m = multiplet, br = broad. Coupling constants (*J*) are in hertz. High-resolution mass spectra were recorded on a Micromass LCT (ESI–TOF–MS).

Silyl ether 14. To a suspension of magnesium turnings (2.14 g, 88.0 mmol) in THF (130 mL) was added 4-bromo-1-butene (8.9 mL, 88.0 mmol) at 0 °C. After the mixture was stirred for 30 min at the same temperature, the mixture was stirred for 1 h at 60 °C. The resulting mixture was added to CuI (1.67 g, 8.80 mmol) at −50 °C and the mixture was stirred for 5 min at the same temperature. To the resulting mixture was added a solution of epoxide **13** (8.40 g, 41.5 mmol) in THF (40 mL + 5.0 mL + 5.0 mL) at −78 °C. After the mixture was stirred for 1 h at the same temperature, the mixture was warmed to room temperature over 2 h. The reaction was quenched with saturated aqueous NH_4Cl. The resulting mixture was diluted with hexane/EtOAc (5:1) solution, washed with H_2O and brine, and then dried over Na_2SO_4. Concentration gave the corresponding alcohol (10.7 g), which was used for the next step without further purification.

To a solution of the alcohol obtained above (10.7 g) in CH_2Cl_2 (137 mL) were added imidazole (4.47 g, 65.6 mmol), DMAP (1.00 g, 8.19 mmol), and TBSCl (7.42 g, 49.2 mmol) at room temperature. After the mixture was stirred for 3 h, to the mixture were added additional imidazole (1.96 g, 28.8 mmol), DMAP (500 mg, 4.09 mmol), and TBSCl (2.26 g, 14.9 mmol) at the same temperature. After the mixture was stirred for 13 h at the same temperature, the reaction was quenched with MeOH. The mixture was diluted with hexane/EtOAc (7:1) solution, washed with H_2O and brine, and then dried over Na_2SO_4. Concentration and column chromatography (hexane, hexane/EtOAc = 50:1) gave silyl ether **14** (14.1 g, 91% in two steps): colorless oil; R_f = 0.80

(hexane/EtOAc = 4:1); $[\alpha]^{24}_{D}$ +8.2 (*c* 1.00, $CHCl_3$); IR (neat) 2929, 2858, 1641 cm^{-1}; ^1H NMR (400 MHz, $CDCl_3$) δ 5.80 (ddt, *J* = 17.1, 10.2, 6.6 Hz, 1H), 5.00 (d, *J* = 17.1 Hz, 1H), 4.94 (d, *J* = 10.2 Hz, 1H), 3.84–3.78 (m, 1H), 3.66 (td, *J* = 6.6, 1.2 Hz, 2H), 2.07–2.02 (m, 2H), 1.65 (q, *J* = 6.6 Hz, 2H), 1.50–1.39 (m, 4H), 0.89 (s, 9H), 0.89 (s, 9H), 0.05 (s, 12H); ^{13}C NMR (100 MHz, $CDCl_3$) δ 138.8, 114.3, 69.2, 60.0, 40.2, 36.9, 33.9, 26.0, 26.0, 24.5, 18.3, 18.2, −4.3, −4.5, −5.2; HRMS (ESI–TOF) calcd for $C_{20}H_{44}O_2Si_2Na$ $[M + Na]^+$ 395.2778, found 395.2780.

Epoxide 15. To a mixture of alkene **14** (36.0 mg, 96.6 μmol) and $NaHCO_3$ (21.0 mg, 0.245 mmol) in CH_2Cl_2 (1.0 mL) was added *m*-CPBA (ca. 70%, 33.0 mg, 0.134 mmol) at 0 °C. After the mixture was stirred for 1 h at the same temperature, the mixture was stirred for 5 h at room temperature. The reaction was quenched with saturated aqueous $NaHCO_3$. The mixture was diluted with Et_2O, washed with H_2O and brine, and then dried over Na_2SO_4. Concentration and column chromatography (hexane/EtOAc = 50:1, 20:1) gave epoxide **15** (34.4 mg, 92%): colorless oil; R_f = 0.43 (hexane/EtOAc = 10:1); IR (neat) 2929, 2857 cm^{-1}; ^1H NMR (400 MHz, $CDCl_3$) δ 3.84–3.81 (m, 1H), 3.66 (t, *J* = 6.6 Hz, 2H), 2.92–2.88 (m, 1H), 2.74 (t, *J* = 4.8 Hz, 1H), 2.46 (dd, *J* = 4.8, 2.6 Hz, 1H), 1.70–1.63 (m, 2H), 1.57–1.45 (m, 6H), 0.89 (s, 9H), 0.89 (s, 9H), 0.05 (s, 3H), 0.05 (s, 3H) 0.05 (s, 6H); ^{13}C NMR (100 MHz, $CDCl_3$) δ 69.1, 59.9, 52.3, 47.1, 40.1, 40.1, 37.2, 37.2, 32.7, 32.7, 26.0, 26.0, 21.7, 21.6, 18.3, 18.2, −4.3, −4.4, −5.2; HRMS (ESI–TOF) calcd for $C_{20}H_{44}O_3Si_2Na$ $[M + Na]^+$ 411.2727, found 411.2732.

Alcohol 17. To a solution of alkyne **16** (1.63 g, 4.90 mmol) in THF (49 mL) was added *n*-BuLi (2.76 M solution in hexane, 1.8 mL, 4.90 mmol) at −78 °C. After the mixture was stirred for 30 min at the same temperature, to the mixture was added $BF{\cdot}OEt_2$ (0.62 mL, 4.90 mmol) at −78 °C. After the mixture was stirred for 30 min at the same temperature, to the resulting solution was added a solution of epoxide **15** (1.01 g, 2.60 mmol) in THF (20 mL + 3.0 mL + 3.0 mL) at −78 °C. The mixture was stirred for 20 min at the same temperature and the mixture was warmed to 0 °C. After the mixture was stirred for 30 min at the same temperature, the reaction was quenched with saturated aqueous NH_4Cl. The resulting mixture was diluted with EtOAc, washed with H_2O and brine, and then dried over Na_2SO_4. Concentration and column chromatography (hexane/EtOAc = 20:1, 5:1) gave alcohol **17** (1.73 g, 92%) and alkyne **16** (530 mg, 33% recovery). Alcohol **17**: colorless oil; R_f = 0.26 (hexane/EtOAc = 10:1); IR (neat) 3464, 2929, 2857 cm^{-1}; ^1H NMR (400 MHz, $CDCl_3$) δ 7.35–7.25 (m, 5H), 4.50 (s, 2H), 4.35 (t, *J* = 6.6 Hz, 1H), 3.83–3.80 (m, 1H), 3.74–3.62 (m, 3H), 3.48 (t, *J* = 6.6 Hz, 2H), 2.43 (ddd, *J* = 16.6, 4.6, 2.0 Hz, 1H), 2.32 (ddt, *J* = 16.6, 6.8, 2.0 Hz, 1H), 1.83 (brs, 1H),

1.71–1.61 (m, 6H), 1.55–1.43 (m, 8H), 0.91 (s, 9H), 0.90 (s, 9H), 0.89 (s, 9H), 0.12 (s, 3H), 0.10 (s, 3H) 0.05 (s, 12H); ^{13}C NMR (100 MHz, $CDCl_3$) δ 138.6, 128.2, 127.5, 127.4, 84.7, 80.5, 72.9, 70.3, 70.0, 70.0, 69.2, 63.1, 60.0, 40.1, 40.1, 38.8, 37.4, 37.4, 36.5, 29.5, 27.8, 27.8, 26.0, 26.0, 25.9, 22.1, 21.3, 21.3, 18.3, 18.3, 18.2, −4.3, −4.3, –4.4, –4.8, −5.2; HRMS (ESI–TOF) calcd for $C_{40}H_{76}O_5Si_3Na$ $[M + Na]^+$ 743.4898, found 743.4902.

Spiroacetal 19. A mixture of alkyne **17** (35.0 mg, 48.5 μmol) and 10% Pd/C (4.9 mg) in EtOAc (0.5 mL) and Et_3N (50 μL) was stirred for 1 h under H_2 atmosphere at room temperature. The catalyst was filtered off and the mixture was washed with EtOAc. Concentration gave the corresponding alkane (36.9 mg), which was used for the next step without further purification.

To a solution of the alcohol obtained above (36.9 mg) in CH_2Cl_2 (0.5 mL) were added MS 4 Å (10.0 mg), TPAP (0.9 mg, 2.5 μmol), and NMO (30.2 mg, 0.258 mmol) at room temperature. The mixture was stirred for 1 h at the same temperature. Short column chromatography (hexane/EtOAc = 7:1) gave ketone **18** (37.8 mg), which was used for the next step without further purification.

To a solution of ketone **18** obtained above (37.8 mg) in MeOH (0.5 mL) was added CSA (2.4 mg, 10.5 μmol) at room temperature. After the mixture was stirred for 40 min, the reaction was quenched with Et_3N. Concentration and column chromatography (hexane/EtOAc = 20:1, 8:1, 5:1) gave spiroacetal **19** (16.8 mg, 95% in three steps): colorless oil; R_f = 0.43 (hexane/EtOAc = 2:1); $[\alpha]^{24}_D$ +50.5 (*c* 1.00, $CHCl_3$); IR (neat) 3458, 2937, 2866 cm^{-1}; ^1H NMR (400 MHz, C_6D_6) δ 7.31 (d, *J* = 7.6 Hz, 2H), 7.20 (t, *J* = 7.6 Hz, 2H), 7.10 (t, *J* = 7.6 Hz, 1H), 4.34 (s, 2H), 3.89–3.72 (m, 3H), 3.68–3.62 (m, 1H), 3.40–3.32 (m, 2H), 2.58 (brs, 1H), 2.05–1.93 (m, 1H), 1.92–1.80 (m, 1H), 1.77–1.50 (m, 8H), 1.45–1.20 (m, 8H), 1.18–1.03 (m, 2H); ^{13}C NMR (100 MHz, C_6D_6) δ 139.5, 128.5, 127.9, 127.5, 96.1, 73.0, 70.6, 69.7, 69.3, 61.4, 39.0, 36.8, 36.0, 35.8, 31.7, 31.6, 30.5, 23.1, 19.7, 19.3; HRMS (ESI–TOF) calcd for $C_{22}H_{34}O_4Na$ $[M + Na]^+$ 385.2355, found 385.2359.

Aldehyde 20. To a solution of alcohol **19** (55.5 mg, 0.153 mmol) in CH_2Cl_2 (1.2 mL) and DMSO (0.4 mL) were added Et_3N (0.11 mL, 0.765 mmol) and SO_3·pyr (97.4 mg, 0.612 mmol) at 0 °C. After the mixture was stirred for 4 h at the same temperature, the reaction was quenched with saturated aqueous NH_4Cl. The mixture was diluted with EtOAc, washed with H_2O and brine, and then dried over Na_2SO_4. Concentration and column chromatography (hexane/EtOAc = 20:1, 10:1) gave aldehyde **20** (52.7 mg, 95%): colorless oil; R_f = 0.71 (hexane/EtOAc = 2:1); $[\alpha]^{27}_D$ +39.0 (*c* 1.00, $CHCl_3$); IR

(neat) 2937, 2866, 1728 cm^{-1}; ^{1}H NMR (400 MHz, $CDCl_3$) δ 9.82 (dd, J = 3.0, 1.9 Hz, 1H), 7.37–7.24 (m, 5H), 4.51 (s, 2H), 4.17–4.11 (m, 1H), 3.55–3.52 (m, 1H), 3.50 (t, J = 6.4 Hz, 2H), 2.54 (ddd, J = 15.9, 9.0, 3.0 Hz, 1H), 2.39 (ddd, J = 15.9, 7.9, 1.9 Hz, 1H), 2.00–1.88 (m, 1H), 1.83–1.71 (m, 1H), 1.71–1.09 (m, 16H); ^{13}C NMR (100 MHz, $CDCl_3$) δ 201.4, 138.7, 128.2, 127.5, 127.4, 96.1, 72.8, 70.4, 69.4, 64.9, 49.8, 36.3, 35.4, 35.3, 31.2, 31.0, 30.0, 22.6, 18.8, 18.7; HRMS (ESI–TOF) calcd for $C_{22}H_{32}O_4Na$ [M + Na]$^+$ 383.2198, found 383.2196.

Sulfone 23. To a solution of alcohol **22** (125 mg, 0.365 mmol) in THF (3.5 mL) were added 1-phenyl-1*H*-tetrazole-5-thiol (98.0 mg, 0.560 mmol), DEAD (40% solution in toluene, 0.25 mL, 0.560 mmol), and PPh_3 (147 mg, 0.560 mmol) at 0 °C. After the mixture was stirred for 10 min at the same temperature, the reaction was quenched with H_2O. The mixture was diluted with EtOAc, washed with H_2O and brine, and then dried over Na_2SO_4. Concentration and column chromatography (hexane/EtOAc = 10:1) gave the corresponding sulfide (168 mg), which was used for the next step without further purification.

To a solution of the sulfide obtained above (168 mg) in EtOH (3.3 mL) were added $(NH_4)_6Mo_7O_{24}\cdot 4H_2O$ (206 mg, 0.167 mmol) and 30% H_2O_2 (0.28 mL, 2.67 mmol) at 0 °C. After the mixture was stirred for 12 h at 40 °C, the reaction was quenched with saturated aqueous Na_2SO_3 at 0 °C. The mixture was diluted with EtOAc, washed with H_2O and brine, and then dried over Na_2SO_4. Concentration and column chromatography (hexane/EtOAc = 10:1) gave sulfone **23** (135 mg, 69% in two steps): colorless oil; R_f = 0.42 (hexane/EtOAc = 4:1); $[\alpha]^{27}_{D}$ −0.62 (c 0.52, $CHCl_3$); IR (neat) 2928 cm^{-1}; ^{1}H NMR (400 MHz, $CDCl_3$) δ 7.68–7.57 (m, 9H), 7.44–7.35 (m, 6H), 3.96 (dd, J = 14.6, 4.6 Hz, 1H), 3.78–3.69 (m, 2H), 3.65 (dd, J = 14.6, 8.1 Hz, 1H), 2.68–2.56 (m, 1H), 1.87–1.79 (m, 1H), 1.65–1.57 (m, 1H), 1.15 (d, J = 6.8 Hz, 3H), 1.03 (s, 9H); ^{13}C NMR (100 MHz, $CDCl_3$) δ 154.0, 135.5, 133.4, 133.1, 131.3, 129.6, 129.6, 127.7, 125.1, 62.0, 61.0, 38.8, 26.9, 25.9, 19.6, 19.2.

Sulfone 24. To a solution of silyl ether **23** (210 mg, 0.393 mmol) in MeOH (2.1 mL) was added CSA (36.5 mg, 0.157 mmol) at room temperature. After the mixture was stirred for 6 h at 40 °C, the reaction was quenched with Et_3N. Concentration and column chromatography (hexane/EtOAc = 3:1, 1:1) gave the corresponding alcohol (112 mg), which was used for the next step without further purification.

To a solution of the alcohol obtained above (112 mg) in CH_2Cl_2 (1.9 mL) were added imidazole (36.0 mg, 0.529 mmol) and TBSCl (68.4 mg, 0.454 mmol) at room temperature. After the mixture was stirred for 30 min at the same temperature, the

reaction was quenched with saturated aqueous $NaHCO_3$. The mixture was diluted with EtOAc, washed with H_2O and brine, and then dried over Na_2SO_4. Concentration and column chromatography (hexane, hexane/EtOAc = 3:1) gave sulfone **24** (148 mg, 92% in two steps): colorless oil; R_f = 0.49 (hexane/EtOAc = 4:1); $[\alpha]^{24}_D$ −4.8 (*c* 0.97, $CHCl_3$); IR (neat) 2929, 2857 cm^{-1}; ^1H NMR (400 MHz, $CDCl_3$) δ 7.70–7.57 (m, 5H), 4.01 (dd, *J* = 14.5, 4.5 Hz, 1H), 3.76–3.66 (m, 2H), 3.64 (dd, *J* = 14.5, 8.3 Hz, 1H), 2.60–2.49 (m, 1H), 1.81–1.73 (m, 1H), 1.65–1.57 (m, 1H), 1.19 (d, *J* = 6.8 Hz, 3H), 0.87 (s, 9H), 0.05 (s, 6H); ^{13}C NMR (100 MHz, $CDCl_3$) δ 154.0, 133.1, 131.3, 129.6, 125.1, 62.0, 60.2, 38.9, 26.1, 25.9, 19.7, 18.2, −5.3, −5.4.

Alkene (*E*)-26. LDA was prepared by adding *n*-BuLi (1.57 M solution in hexane, 0.54 mL, 0.845 mmol) to a solution of diisopropylamine (0.12 ml, 0.845 mmol) in THF (0.6 mL) at 0 °C. The solution was stirred for 30 min at 0 °C.

To a solution of sulfone **24** (47.2 mg, 0.115 mmol) in THF (0.5 mL) was added LDA (0.74 M solution in THF, 0.14 mL, 0.104 mmol) at −78 °C. After the mixture was stirred for 30 min at the same temperature, to the mixture was added a solution of aldehyde **20** (16.0 mg, 44.4 μmol) in THF (0.5 mL + 0.5 mL). After the mixture was stirred for 30 min at −78 °C, the reaction was quenched with saturated aqueous NH_4Cl. The mixture was diluted with EtOAc, washed with H_2O and brine, and then dried over Na_2SO_4. Concentration and column chromatography (hexane/EtOAc = 30:1, 20:1) gave alkenes (*E*)- and (*Z*)-**26** (20.8 mg, 86%, *E*:*Z* = 5.0:1) as a diastereomeric mixture and sulfone **24** (28.3 mg, 60% recovery). Alkenes (*E*)- and (*Z*)-**26**: colorless oil; R_f = 0.74 (hexane/EtOAc = 4:1); IR (neat) 2930, 2857 cm^{-1}; ^1H NMR (400 MHz, C_6D_6) δ 7.31 (d, *J* = 7.4 Hz, 2H), 7.19 (t, *J* = 7.4 Hz, 2H), 7.10 (t, *J* = 7.4 Hz, 1H), 5.71–5.60 (m, 1H), 5.41 (dd, *J* = 15.3, 7.8 Hz, 0.83H), 5.29 (t, *J* = 10.7 Hz, 0.17H), 4.35 (s, 1.67H), 4.34 (s, 0.33H), 3.78–3.66 (m, 2H), 3.64–3.59 (m, 2H), 3.39–3.34 (m, 2H), 2.44–2.30 (m, 2H), 2.22–2.00 (m, 3H), 1.81–1.11 (m, 18H), 1.03 (d, *J* = 6.8 Hz, 3H), 1.00 (s, 1.50H), 0.99 (s, 7.50H), 0.08–0.07 (m, 6H); ^{13}C NMR (100 MHz, C_6D_6) δ 139.6, 138.3, 137.1, 133.4, 130.7, 129.0, 128.5, 127.9, 127.6, 127.5, 125.9, 125.8, 96.0, 73.0, 70.6, 69.6, 69.4, 69.2, 69.1, 68.1, 61.5, 61.5, 41.0, 40.6, 40.2, 39.3, 36.9, 36.2, 36.1, 35.1, 33.8, 32.4, 31.9, 31.9, 31.5, 31.4, 30.9, 30.6, 30.3, 29.9, 29.6, 29.4, 28.7, 26.3, 24.3, 23.4, 23.2, 23.1, 21.6, 21.2,19.6, 19.5, 18.6, 14.5, 14.4, 11.3, −4.9, −5.0.

Diol 27. To a solution of alkenes (*E*)- and (*Z*)-**26** (29.3 mg, 53.8 μmol, *E*:*Z* = 5.0:1) in *t*-BuOH (0.5 mL) and H_2O (0.5 mL) were added $MeSO_2NH_2$ (5.1 mg, 53.8 μmol) and AD-mix-β (75.3 mg) at 0 °C. After the mixture was stirred for 15 min at the same temperature, the mixture was stirred for 20 h at room temperature. To the mixture were

added $MeSO_2NH_2$ (5.1 mg, 53.8 μmol) and AD-mix-β (75.3 mg) at room temperature. After the mixture was stirred for 16 h at the same temperature, the reaction was quenched with saturated aqueous $NaHCO_3$. The mixture was diluted with EtOAc, washed with H_2O and brine, and then dried over Na_2SO_4. Concentration and column chromatography (hexane/EtOAc = 8:1, 4:1) gave diol **27** (18.7 mg, 72% from (*E*)-**26**) and (*E*)- and (*Z*)-**26** (6.6 mg, *E*:*Z* = 1:10). Diol **27**: colorless oil; R_f = 0.23 (hexane/EtOAc = 4:1); $[\alpha]^{25}_{D}$ +22.1 (*c* 0.24, $CHCl_3$); IR (neat) 3435, 2929, 2856 cm^{-1}; ^1H NMR (400 MHz, $CDCl_3$) δ 7.34–7.24 (m, 5H), 4.50 (s, 2H), 3.99–3.91 (m, 2H), 3.78–3.73 (m, 1H), 3.68–3.61 (m, 2H), 3.52–3.46 (m, 3H), 3.18–3.15 (m, 1H), 2.86 (d, *J* = 5.4 Hz, 1H), 1.97–1.11 (m, 23H), 1.00 (d, *J* = 6.8 Hz, 3H), 0.90 (s, 9H), 0.08 (s, 3H), 0.08 (s, 3H); ^{13}C NMR (100 MHz, $CDCl_3$) δ 138.6, 128.2, 127.5, 127.4, 95.9, 78.7, 72.8, 70.4, 69.1, 68.4, 66.5, 61.2, 40.1, 36.4, 35.6, 35.0, 33.2, 31.4, 31.2, 29.9, 26.0, 22.5, 19.1, 18.9, 18.3, 17.4, −5.4.

Stereochemical confirmation at the C93 and C94 positions of 27. Treatment of the diol **27** with (*R*)- or (*S*)-MTPACl/pyridine/DMAP provided (*S*)-MTPA ester **S1** and (*R*)-MTPA ester **S2**, respectively (Scheme S1). The results for calculating the chemical shift differences ($\Delta\delta_{S-R}$) of **S1** and **S2** in $CDCl_3$ are described in Figure S1a. Thus, the absolute configuration at the C93 position of **27** was confirmed by the modified Mosher method. Furthermore, the hydroxy group at the C93 position of **27** was protected to give TBS ether **S3**. Treatment of **S3** with (*R*)- or (*S*)-MTPACl/Et_3N/DMAP provided (*S*)-MTPA ester **S4** and (*R*)-MTPA ester **S5**, respectively. The chemical shift differences ($\Delta\delta_{S-R}$) of **S4** and **S5** in $CDCl_3$ are described in Figure S1b. Thus, the absolute configuration at the C94 position of **S3** was confirmed by the modified Mosher method.

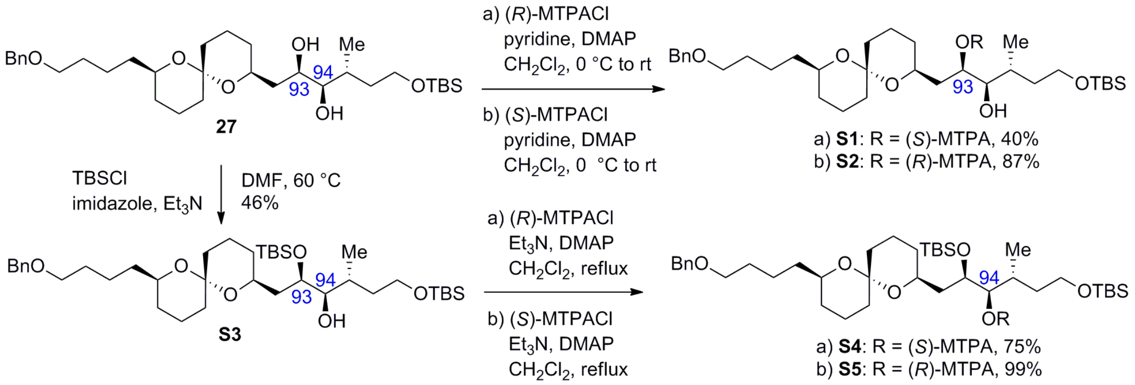

Scheme S1: Derivatization of **27** for the stereochemical confirmation. MTPA = α-methoxy-α-(trifluoromethyl)phenylacetyl.

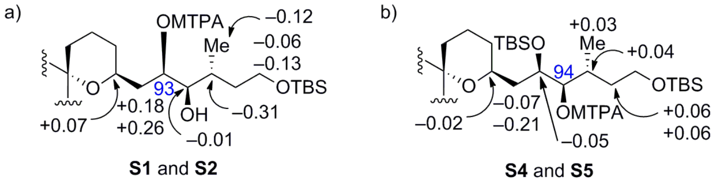

Figure S1: Chemical shift differences ($\Delta\delta_{S-R}$) of **S1**/**S2** (a) and **S4**/**S5** (b).

(*S*)-MTPA Ester S1. To a solution of diol **27** (2.0 mg, 3.45 μmol) in CH_2Cl_2 (0.1 mL) were added DMAP (0.8 mg, 6.90 μmol), pyridine (0.5 μL, 5.52 μmol), and (*R*)-MTPACl (0.8 μL, 4.49 μmol) at 0 °C. After the mixture was stirred for 2 h at the same temperature and for 2 h at room temperature, the reaction was quenched with saturated aqueous NH_4Cl. The mixture was diluted with EtOAc, washed with H_2O and brine, and then dried over Na_2SO_4. Concentration and column chromatography (hexane/EtOAc = 20:1, 10:1) gave (*S*)-MTPA ester **S1** (1.1 mg, 40%): colorless oil; R_f = 0.40 (hexane/EtOAc = 4:1); ^{1}H NMR (400 MHz, $CDCl_3$) δ 7.56–7.55 (m, 2H), 7.36–7.30 (m, 8H), 5.32–5.27 (m, 1H), 4.48 (s, 2H), 3.73–3.66 (m, 1H), 3.65–3.59 (m, 1H), 3.57–3.47 (m, 2H), 3.54 (s, 3H), 3.45–3.42 (m, 3H), 2.08–2.00 (m, 1H), 1.90–1.07 (m, 23H), 0.86 (s, 9H), 0.80 (d, *J* = 6.6 Hz, 3H), 0.02 (s, 3H), 0.01 (s, 3H).

(*R*)-MTPA Ester S2. To a solution of diol **27** (0.5 mg, 0.86 μmol) in CH_2Cl_2 (0.1 mL) were added DMAP (0.2 mg, 0.86 μmol), pyridine (0.2 μL, 2.76 μmol), and (*S*)-MTPACl (0.4 μL, 2.00 μmol) at 0 °C. After the mixture was stirred for 17 h at the same temperature and for 4 h at room temperature, the reaction was quenched with saturated aqueous NH_4Cl. The mixture was diluted with EtOAc, washed with H_2O and brine, and then dried over Na_2SO_4. Concentration and column chromatography (hexane/EtOAc = 20:1, 10:1) gave (*R*)-MTPA ester **S2** (0.6 mg, 87%): colorless oil; R_f = 0.40 (hexane/EtOAc = 4:1); ^{1}H NMR (400 MHz, $CDCl_3$) δ 7.56–7.55 (m, 2H), 7.37–7.30 (m, 8H), 5.39–5.35 (m, 1H), 4.47 (s, 2H), 3.72–3.66 (m, 1H), 3.65–3.58 (m, 3H), 3.53 (s, 3H), 3.47–3.43 (m, 3H), 3.19 (d, *J* = 7.6 Hz, 1H), 1.91–1.16 (m, 23H), 0.93 (d, *J* = 6.6 Hz, 3H), 0.87 (s, 9H), 0.05 (s, 6H).

Silyl ether S3. To a solution of diol **27** (2.9 mg, 5.01 μmol) in DMF (0.1 mL) were added imidazole (1.7 mg, 25.1 μmol), Et_3N (3.5 μL, 25.1 μmol), and TBSCl (2.3 mg, 15.0 μmol) at room temperature. After the mixture was stirred for 6 h at 60 °C, the reaction was quenched with saturated aqueous $NaHCO_3$. The mixture was diluted with Et_2O, washed with H_2O and brine, and then dried over Na_2SO_4. Concentration and column chromatography (hexane/EtOAc = 25:1, 15:1) gave silyl ether **S3** (1.6 mg,

46%): colorless oil; R_f = 0.41 (hexane/EtOAc = 7:1); $[\alpha]^{21}{}_D$ +18.0 (*c* 1.12, $CHCl_3$); IR (neat) 3560, 2933, 2857 cm^{-1}; ^1H NMR (400 MHz, C_6D_6) δ 7.33 (d, *J* = 7.3 Hz, 2H), 7.21 (t, *J* = 7.3 Hz, 2H), 7.11 (t, *J* = 7.3 Hz, 1H), 4.37 (s, 2H), 4.14–4.12 (m, 1H), 3.92–3.66 (m, 4H), 3.55 (t, *J* = 8.8 Hz, 1H), 3.40 (t, *J* = 6.1 Hz, 2H), 2.52–2.36 (m, 3H), 2.11–1.95 (m, 3H), 1.82–1.07 (m, 18H), 1.05 (d, *J* = 6.6 Hz, 3H), 1.02 (s, 9H), 0.96 (s, 9H), 0.14 (s, 3H), 0.13 (s, 3H), 0.11 (s, 3H), 0.11 (s, 3H); ^{13}C NMR (100 MHz, C_6D_6) δ 139.5, 128.5, 127.6, 127.5, 96.1, 78.1, 73.0, 71.9, 70.6, 69.4, 67.9, 61.9, 42.0, 36.9, 36.9, 36.1, 35.9, 33.4, 32.3, 31.6, 30.6, 26.3, 26.3, 23.3, 19.4, 19.3, 18.6, 18.5, 17.0, –3.4, –4.2, –4.9, –5.0; HRMS (ESI–TOF) calcd for $C_{39}H_{72}O_6Si_2Na$ $[M + Na]^+$ 715.4765, found 715.4765.

(*S*)-MTPA Ester S4. To a solution of alcohol **S3** (1.2 mg, 1.73 μmol) in CH_2Cl_2 (0.1 mL) were added DMAP (0.2 mg, 1.73 μmol), Et_3N (0.4 μL, 2.94 μmol), and (*R*)-MTPACl (0.4 μL, 2.25 μmol) at room temperature. After the mixture was stirred for 2 h at reflux, the reaction was quenched with saturated aqueous NH_4Cl. The mixture was diluted with EtOAc, washed with H_2O and brine, and then dried over Na_2SO_4. Concentration and column chromatography (hexane/EtOAc = 20:1) gave (*S*)-MTPA ester **S4** (1.2 mg, 75%): colorless oil; R_f = 0.32 (hexane/EtOAc = 10:1); ^1H NMR (400 MHz, $CDCl_3$) δ 7.67–7.60 (m, 2H), 7.42–7.21 (m, 8H), 5.05 (dd, *J* = 9.3, 1.4 Hz, 1H), 4.50 (s, 2H), 4.05–4.03 (m, 1H), 3.66–3.49 (m, 4H), 3.55 (s, 3H), 3.47 (t, *J* = 6.6 Hz, 2H), 2.22–2.20 (m, 1H), 1.94–1.79 (m, 2H), 1.75–1.01 (m, 20H), 0.97 (d, *J* = 6.8 Hz, 3H), 0.88 (s, 9H), 0.84 (s, 9H), 0.05 (s, 3H), 0.02 (s, 6H), 0.01 (s, 3H); ^{13}C NMR (100 MHz, $CDCl_3$) δ 166.2, 138.7, 131.8, 129.4, 128.2, 128.0, 127.5, 127.4, 95.9, 82.9, 72.8, 70.4, 70.3, 69.2, 67.4, 60.6, 55.5, 40.6, 36.5, 35.7, 35.5, 35.5, 32.0, 31.0, 30.2, 30.1, 26.0, 25.8, 22.5, 18.9, 18.7, 18.0, 16.1, −4.0, −4.2, −5.3, −5.3; HRMS (ESI–TOF) calcd for $C_{49}H_{79}F_3O_8Si_2Na$ $[M + Na]^+$ 931.5164, found 931.5155.

(*R*)-MTPA Ester S5. To a solution of alcohol **S3** (1.3 mg, 1.88 μmol) in CH_2Cl_2 (0.1 mL) were added DMAP (0.2 mg, 1.73 μmol), Et_3N (0.5 μL, 3.20 μmol), and (*S*)-MTPACl (0.5 μL, 2.44 μmol) at room temperature. After the mixture was stirred for 2 h at reflux, the reaction was quenched with saturated aqueous NH_4Cl. The mixture was diluted with EtOAc, washed with H_2O and brine, and then dried over Na_2SO_4. Concentration and column chromatography (hexane/EtOAc = 20:1) gave (*R*)-MTPA ester **S5** (1.7 mg, 99%): colorless oil; R_f = 0.37 (hexane/EtOAc = 10:1); ^1H NMR (400 MHz, $CDCl_3$) δ 7.67–7.62 (m, 2H), 7.39–7.23 (m, 8H), 4.97 (dd, *J* = 9.0, 1.5 Hz, 1H), 4.50 (s, 2H), 4.10–4.08 (m, 1H), 3.63–3.61 (m, 1H), 3.59 (s, 3H), 3.55–3.43 (m, 3H), 3.47 (t, *J* = 6.6 Hz, 2H), 2.18–2.15 (m, 1H), 1.89–1.81 (m, 1H), 1.79–1.03 (m,

21H), 0.93 (d, *J* = 6.8 Hz, 3H), 0.88 (s, 9H), 0.84 (s, 9H), 0.10 (s, 3H), 0.08 (s, 3H), −0.02 (s, 3H), −0.03 (s, 3H); ^{13}C NMR (100 MHz, $CDCl_3$) δ 166.2, 138.7, 132.1, 129.3, 128.2, 128.1, 127.7, 127.5, 127.4, 95.8, 82.7, 72.8, 70.4, 69.8, 69.2, 67.1, 60.6, 55.6, 41.0, 36.5, 35.7, 35.6, 35.2, 31.9, 31.0, 30.3, 30.1, 26.0, 25.9, 22.3, 18.8, 18.6, 18.3, 18.1, 16.3, −4.0, −4.2, −5.4; HRMS (ESI–TOF) calcd for $C_{49}H_{79}F_3O_8Si_2Na$ $[M + Na]^+$ 931.5164, found 931.5156.

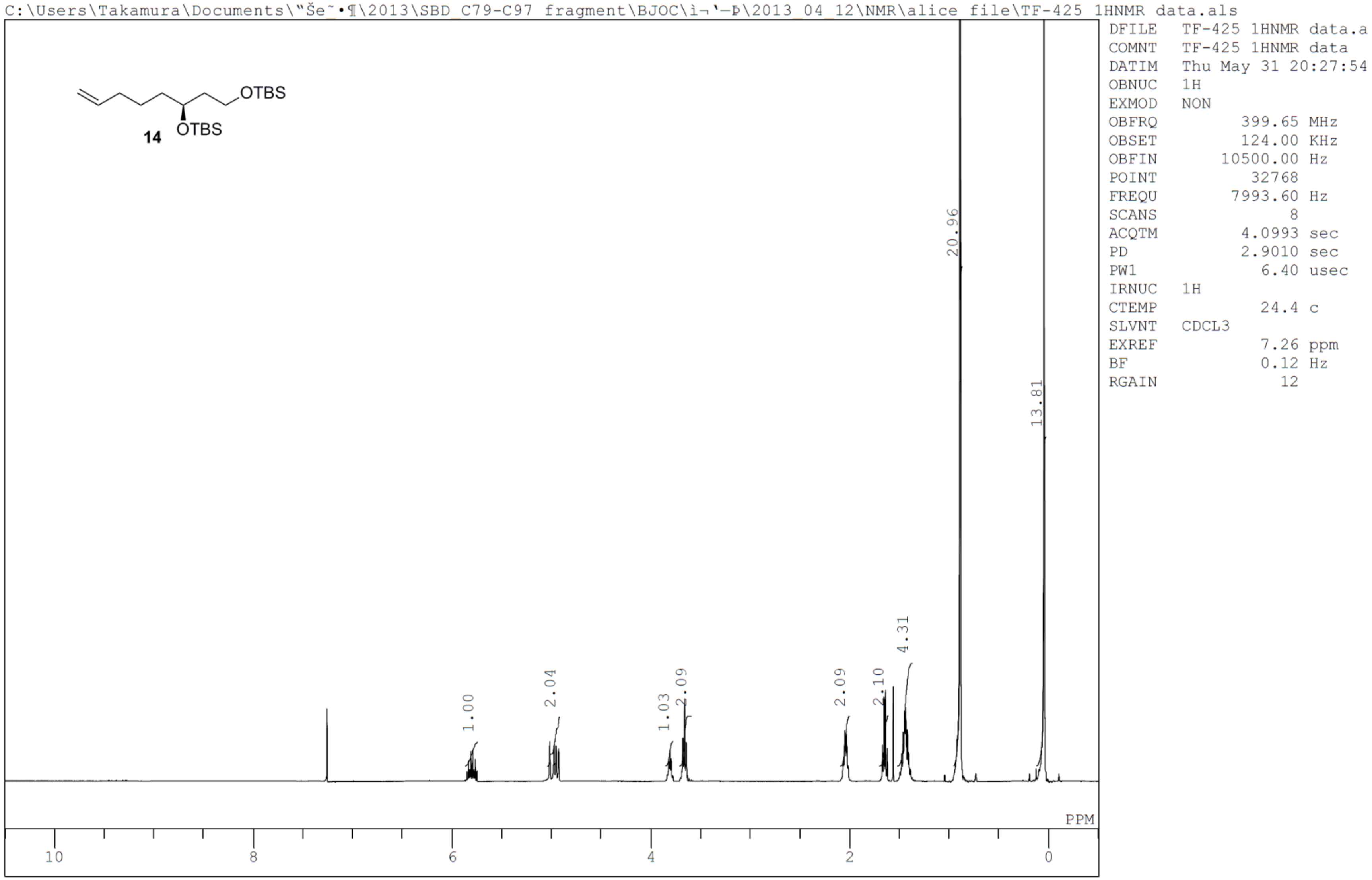
C:\Users\Takamura\Documents\"Še˜•¶\2013\SBD C79-C97 fragment\BJOC\ì¬'—Þ\2013 04 12\NMR\alice file\TF-425 1HNMR data.als
OTBS
OTBS
14
DFILE TF-425 1HNMR data.a
COMNT TF-425 1HNMR data
DATIM Thu May 31 20:27:54
OBNUC 1H
EXMOD NON
OBFRQ 399.65 MHz
OBSET 124.00 KHz
OBFIN 10500.00 Hz
POINT 32768
FREQU 7993.60 Hz
SCANS 8
ACQTM 4.0993 sec
PD 2.9010 sec
PW1 6.40 usec
IRNUC 1H
CTEMP 24.4 c
SLVNT CDCL3
EXREF 7.26 ppm
BF 0.12 Hz
RGAIN 12
20.96
13.81
4.31
2.10
2.09
2.09
1.03
2.04
1.00
PPM
10
8
6
4
2
0

C:\Users\Takamura\Documents\"Še˜•¶\2013\SBD_C79-C97_fragment\BJOC\i¬'−Þ\2013_04_12\NMR\alice file\TF-425 13CNMR data.als

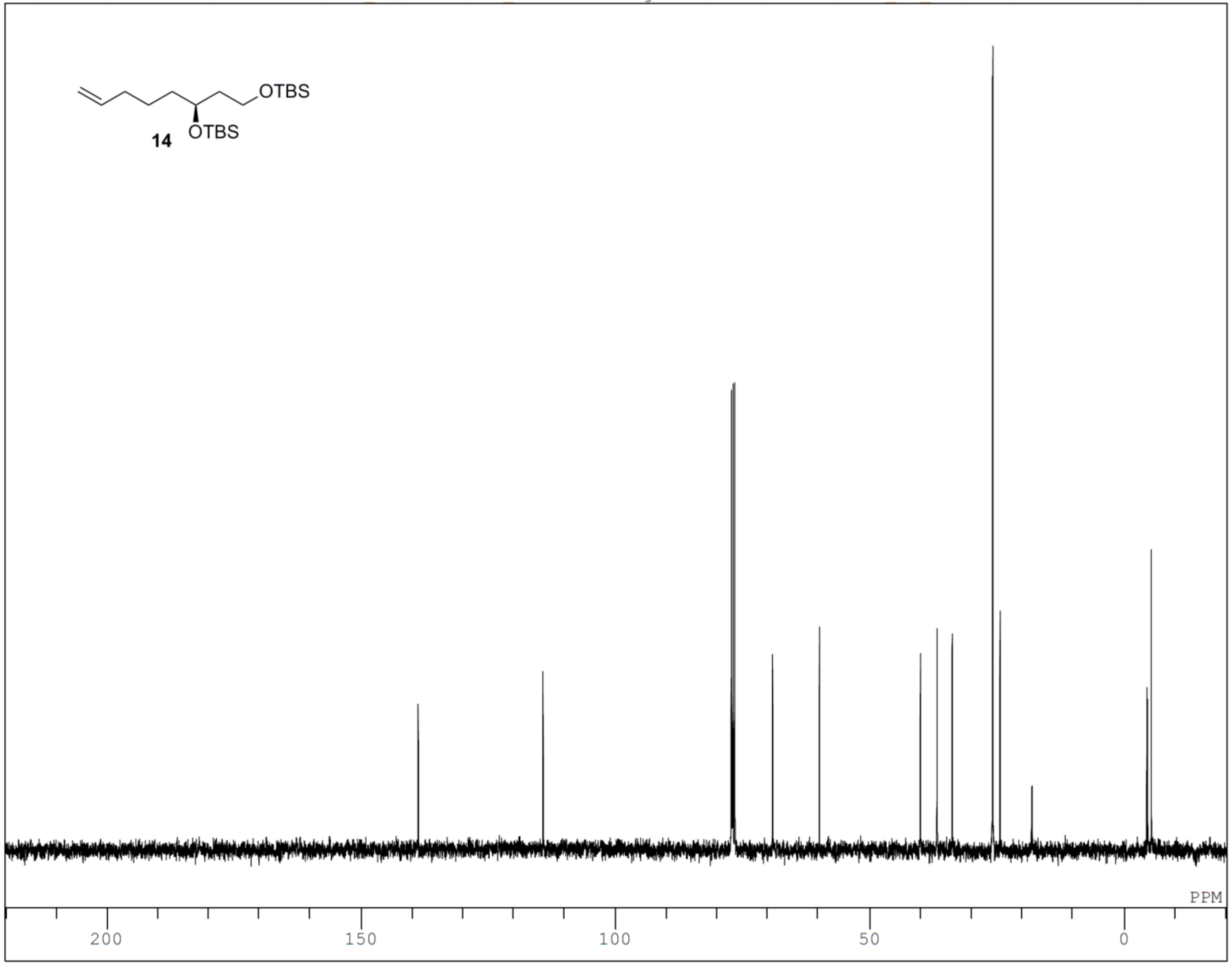

DFILE	TF-425 13CNMR data.
COMNT	TF-425 13CNMR data
DATIM	Thu May 31 20:43:55
OBNUC	13C
EXMOD	BCM
OBFRQ	100.40 MHz
OBSET	125.00 KHz
OBFIN	10500.00 Hz
POINT	32768
FREQU	27173.90 Hz
SCANS	200
ACQTM	1.2059 sec
PD	1.7940 sec
PW1	6.80 usec
IRNUC	1H
CTEMP	27.6 c
SLVNT	CDCL3
EXREF	77.00 ppm
BF	2.00 Hz
RGAIN	23

C:\Users\Takamura\Documents\"Še˜•¶\2013\SBD C79-C97 fragment\BJOC\ì¬'─Þ\2013 04 12\NMR\alice file\TF-426 1HNMR data.als

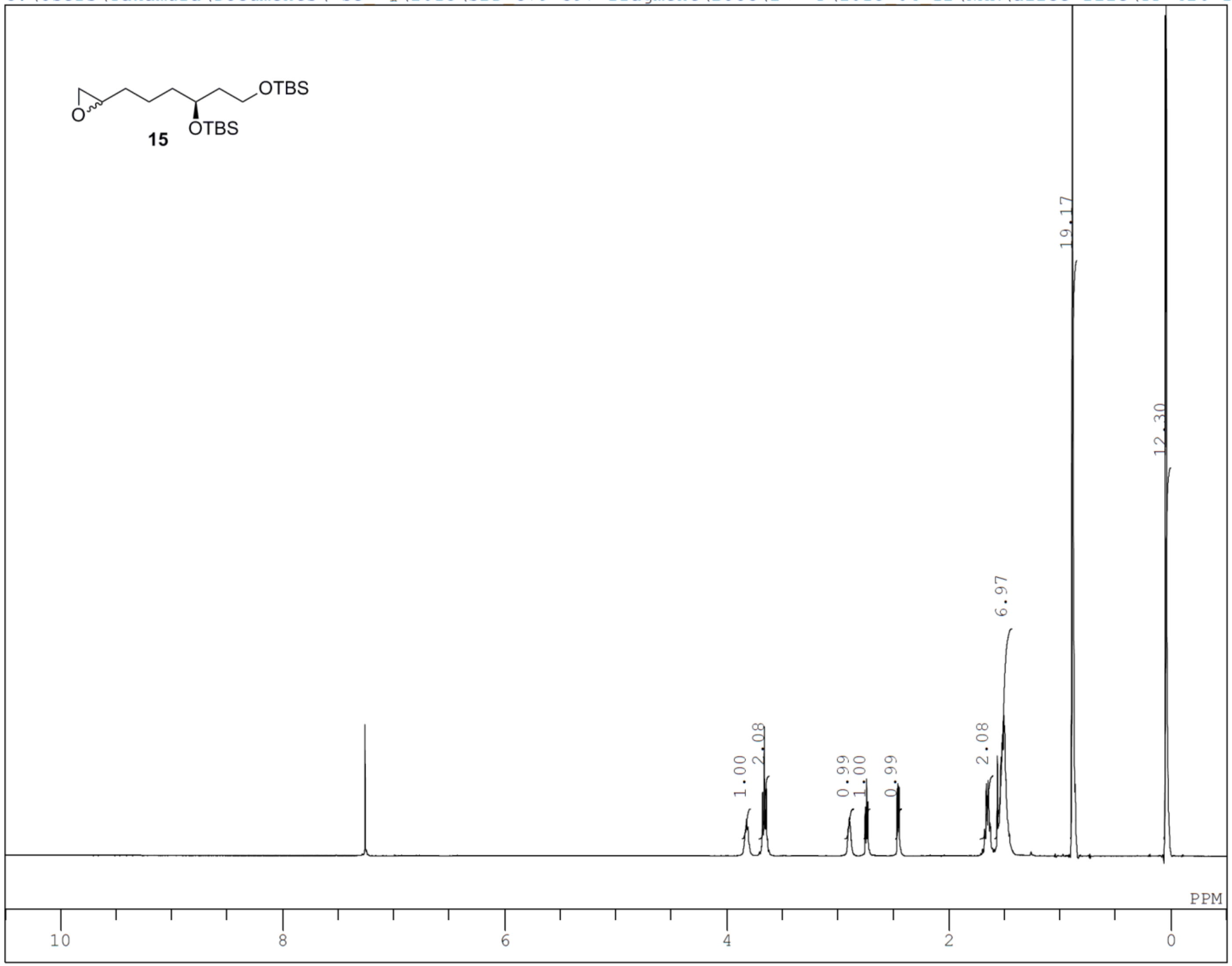

DFILE	TF-426 1HNMR data.a
COMNT	
DATIM	Mon Jun 04 23:45:39
OBNUC	1H
EXMOD	NON
OBFRQ	399.65 MHz
OBSET	124.00 KHz
OBFIN	10500.00 Hz
POINT	32768
FREQU	7993.60 Hz
SCANS	8
ACQTM	4.0993 sec
PD	2.9010 sec
PW1	6.40 usec
IRNUC	1H
CTEMP	25.9 c
SLVNT	CDCL3
EXREF	7.26 ppm
BF	0.12 Hz
RGAIN	12

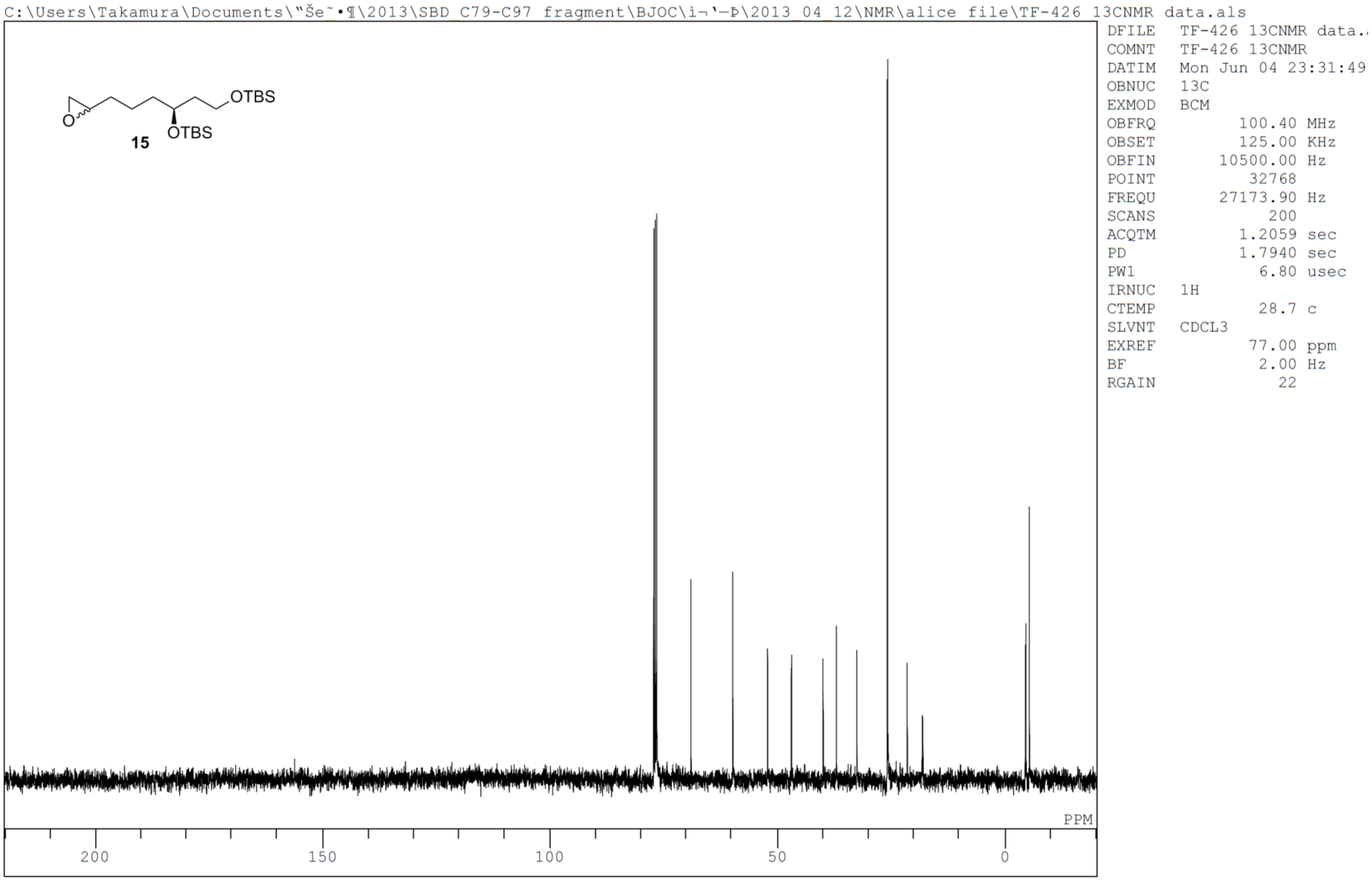
C:\Users\Takamura\Documents\"Še˜•¶\2013\SBD C79-C97 fragment\BJOC\ì¬'−Þ\2013 04 12\NMR\alice file\TF-426 13CNMR data.als
OTBS
OTBS
15
DFILE TF-426 13CNMR data.
COMNT TF-426 13CNMR
DATIM Mon Jun 04 23:31:49
OBNUC 13C
EXMOD BCM
OBFRQ 100.40 MHz
OBSET 125.00 KHz
OBFIN 10500.00 Hz
POINT 32768
FREQU 27173.90 Hz
SCANS 200
ACQTM 1.2059 sec
PD 1.7940 sec
PW1 6.80 usec
IRNUC 1H
CTEMP 28.7 c
SLVNT CDCL3
EXREF 77.00 ppm
BF 2.00 Hz
RGAIN 22
PPM
200
150
100
50
0

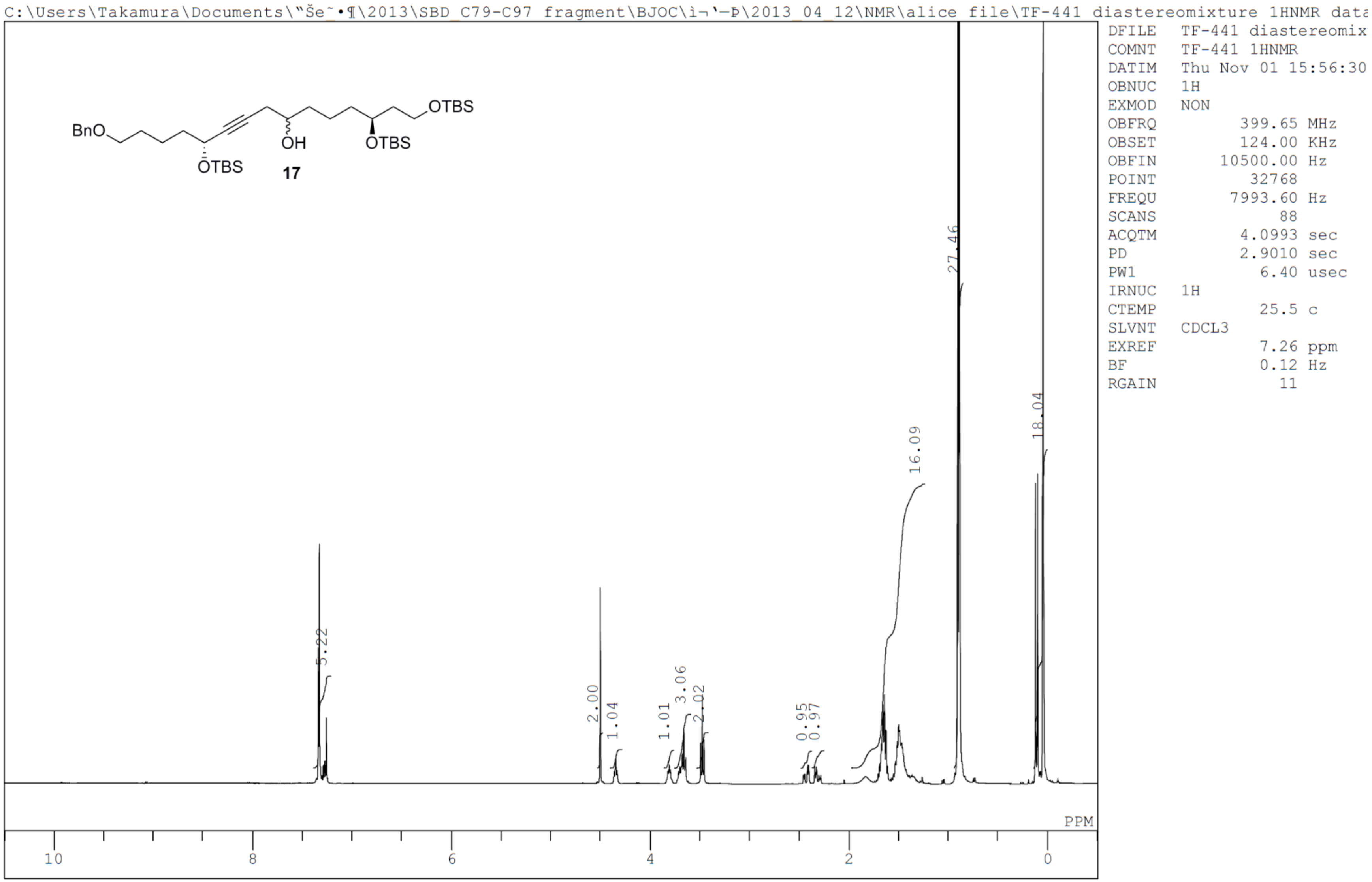
C:\Users\Takamura\Documents\"Še˜•¶\2013\SBD C79-C97 fragment\BJOC\ì¬'—Þ\2013 04 12\NMR\alice file\TF-441 diastereomixture 1HNMR data
BnO
OTBS
OH
OTBS
OTBS
17
5.22
2.00
1.04
1.01
3.06
2.02
0.95
0.97
16.09
27.46
18.04
PPM
10
8
6
4
2
0
DFILE TF-441 diastereomix
COMNT TF-441 1HNMR
DATIM Thu Nov 01 15:56:30
OBNUC 1H
EXMOD NON
OBFRQ 399.65 MHz
OBSET 124.00 KHz
OBFIN 10500.00 Hz
POINT 32768
FREQU 7993.60 Hz
SCANS 88
ACQTM 4.0993 sec
PD 2.9010 sec
PW1 6.40 usec
IRNUC 1H
CTEMP 25.5 c
SLVNT CDCL3
EXREF 7.26 ppm
BF 0.12 Hz
RGAIN 11

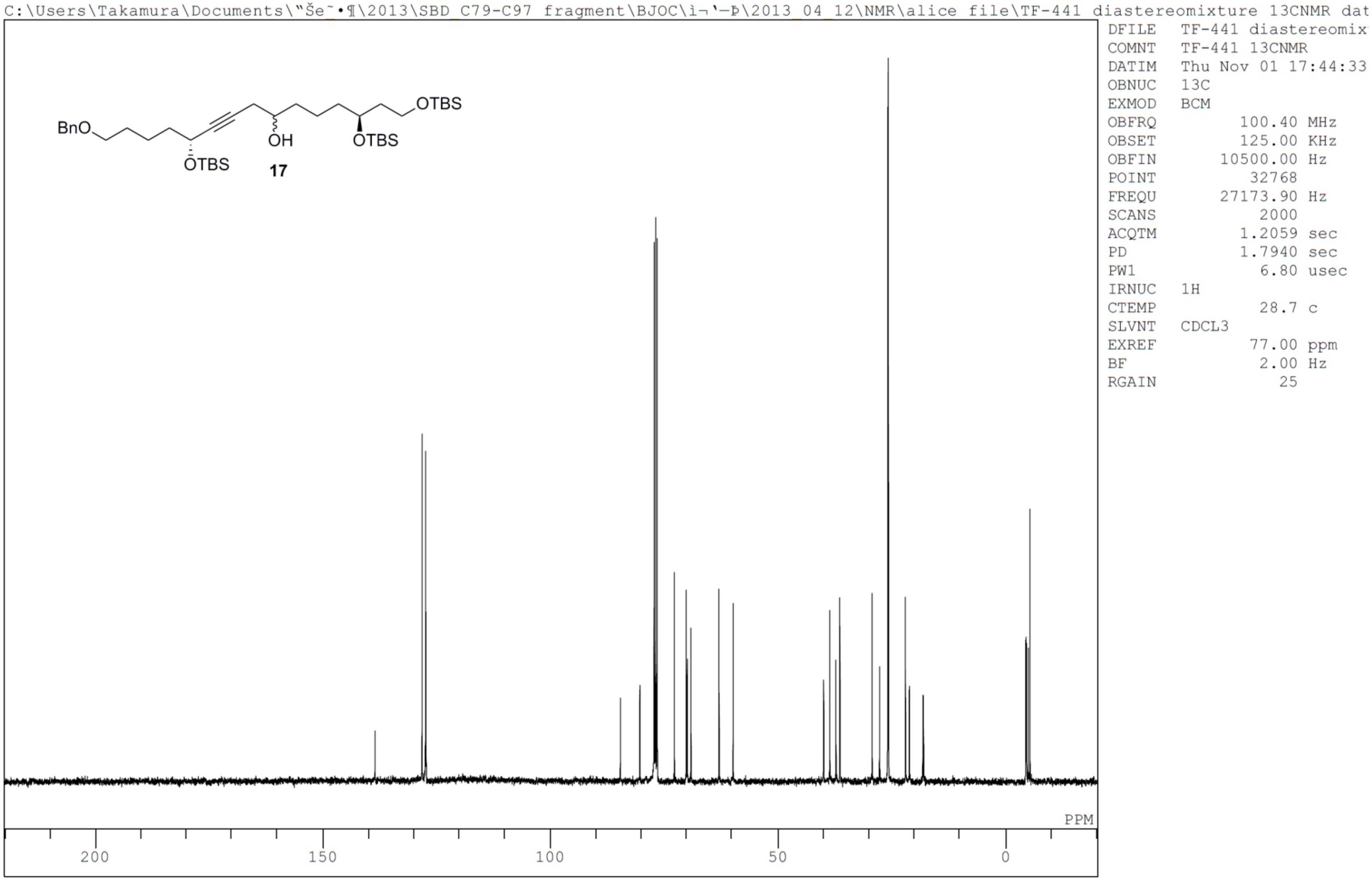
C:\Users\Takamura\Documents\"Še˜•¶\2013\SBD_C79-C97 fragment\BJOC\ì¬'—Þ\2013_04_12\NMR\alice file\TF-441 diastereomixture 13CNMR dat
BnO
OTBS
OH
OTBS
OTBS
17
DFILE TF-441 diastereomix
COMNT TF-441 13CNMR
DATIM Thu Nov 01 17:44:33
OBNUC 13C
EXMOD BCM
OBFRQ 100.40 MHz
OBSET 125.00 KHz
OBFIN 10500.00 Hz
POINT 32768
FREQU 27173.90 Hz
SCANS 2000
ACQTM 1.2059 sec
PD 1.7940 sec
PW1 6.80 usec
IRNUC 1H
CTEMP 28.7 c
SLVNT CDCL3
EXREF 77.00 ppm
BF 2.00 Hz
RGAIN 25
PPM
200
150
100
50
0

C:\Users\Takamura\Documents\"Še˜•¶\2013\SBD_C79-C97 fragment\BJOC\ì¬'–Þ\2013_04_12\NMR\alice file\TF-444 C6D6 1HNMR data.als

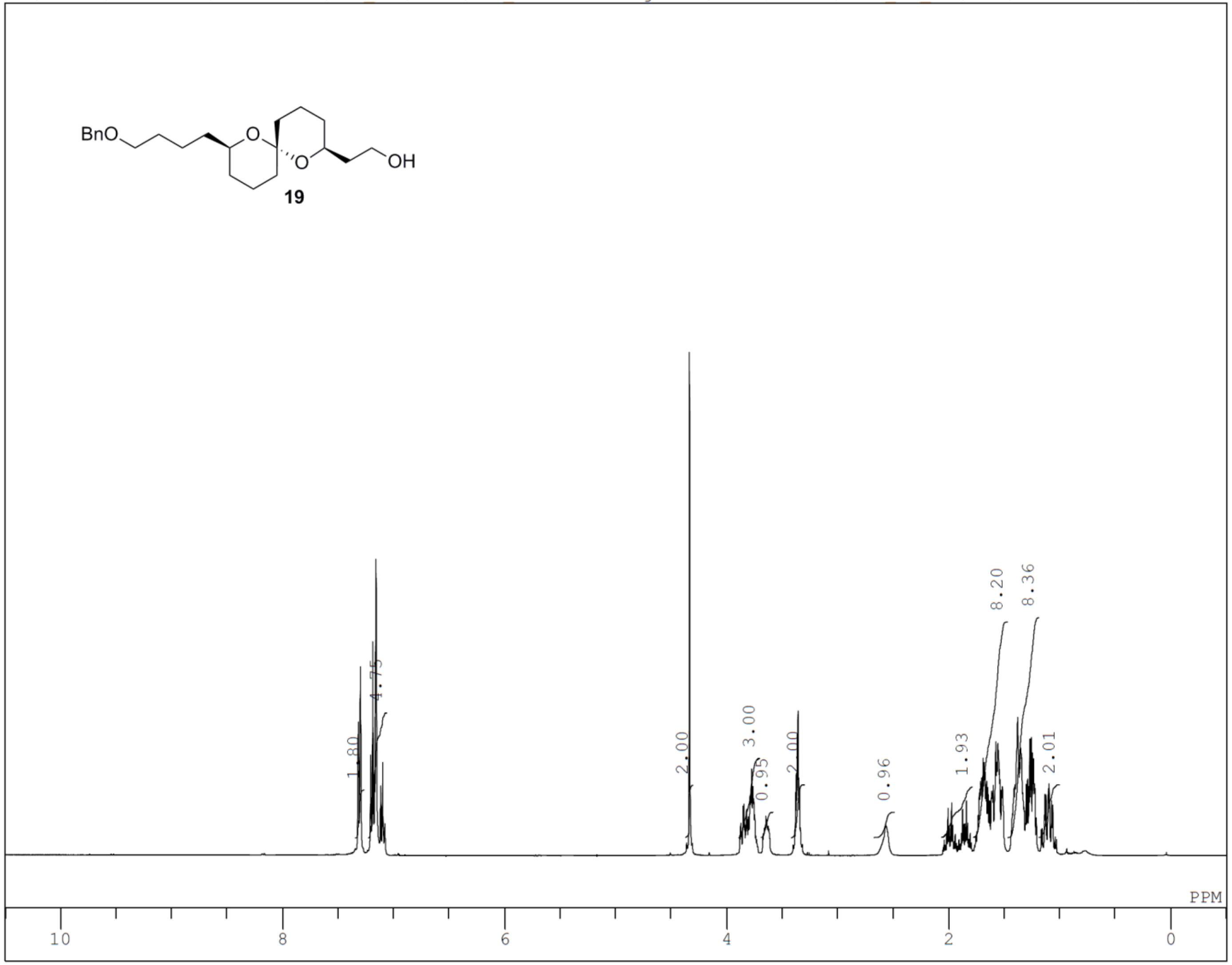

DFILE	TF-444 C6D6 1HNMR d	
COMNT	TF-444 1HNMR	
DATIM	Fri Nov 02 14:48:19	
OBNUC	1H	
EXMOD	NON	
OBFRQ	399.65	MHz
OBSET	124.00	KHz
OBFIN	10500.00	Hz
POINT	32768	
FREQU	7993.60	Hz
SCANS	8	
ACQTM	4.0993	sec
PD	2.9010	sec
PW1	6.40	usec
IRNUC	1H	
CTEMP	25.7	c
SLVNT	C6D6	
EXREF	7.16	ppm
BF	0.12	Hz
RGAIN	12	

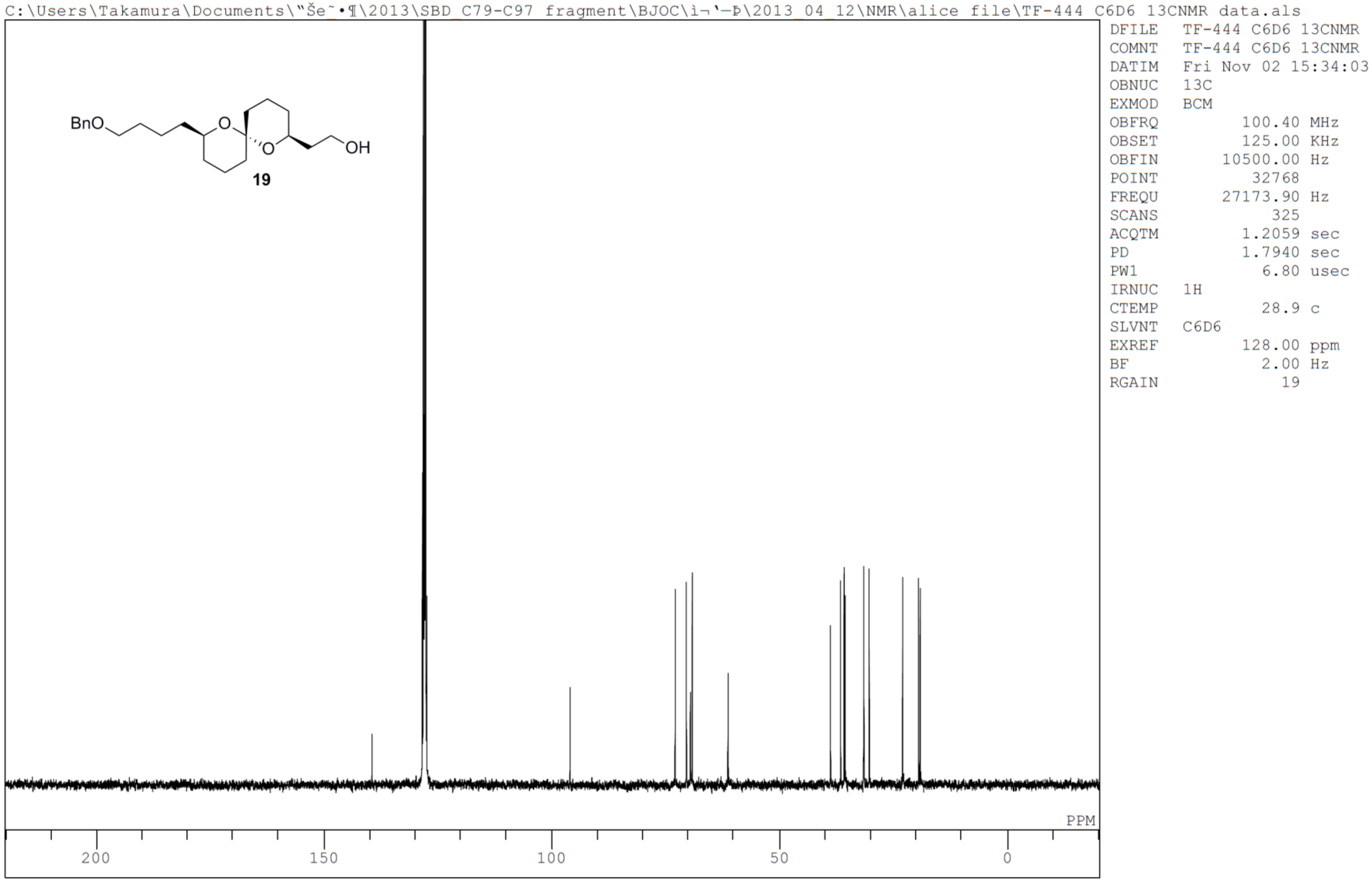
C:\Users\Takamura\Documents\"Še˜•¶\2013\SBD_C79-C97 fragment\BJOC\i¬'–Þ\2013_04_12\NMR\alice file\TF-444 C6D6 13CNMR data.als
BnO
O
O
OH
19
DFILE TF-444 C6D6 13CNMR
COMNT TF-444 C6D6 13CNMR
DATIM Fri Nov 02 15:34:03
OBNUC 13C
EXMOD BCM
OBFRQ 100.40 MHz
OBSET 125.00 KHz
OBFIN 10500.00 Hz
POINT 32768
FREQU 27173.90 Hz
SCANS 325
ACQTM 1.2059 sec
PD 1.7940 sec
PW1 6.80 usec
IRNUC 1H
CTEMP 28.9 c
SLVNT C6D6
EXREF 128.00 ppm
BF 2.00 Hz
RGAIN 19
PPM
200
150
100
50
0

C:\Users\Takamura\Documents\“Še˜•¶\2013\SBD_C79-C97 fragment\BJOC\ì¬'-Þ\2013_04_12\NMR\alice file\TF-531 1HNMR data.als

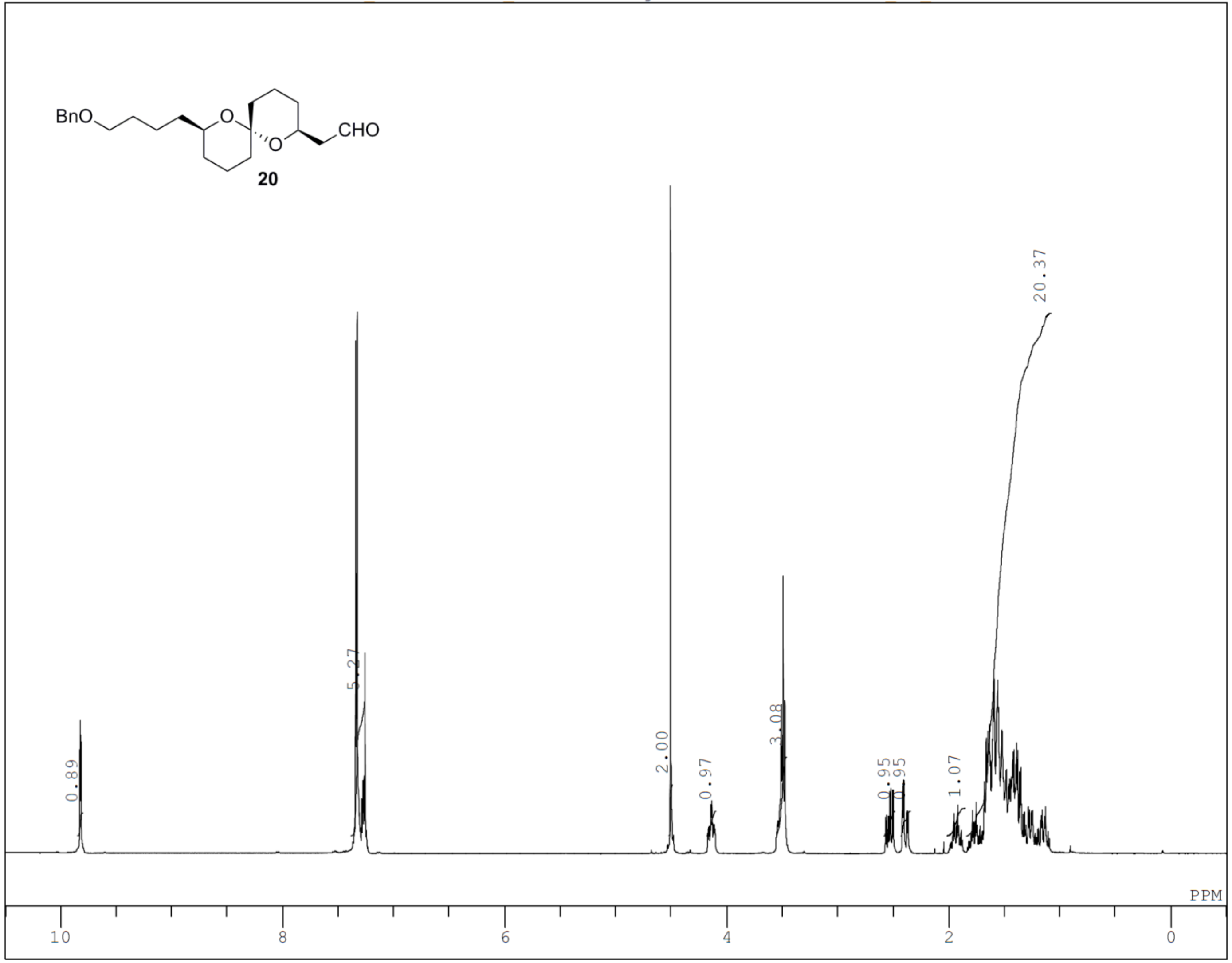

DFILE	TF-531 1HNMR data.a
COMNT	TF-531
DATIM	Mon Sep 10 13:30:32
OBNUC	1H
EXMOD	NON
OBFRQ	399.65 MHz
OBSET	124.00 KHz
OBFIN	10500.00 Hz
POINT	32768
FREQU	7993.60 Hz
SCANS	8
ACQTM	4.0993 sec
PD	2.9010 sec
PW1	6.40 usec
IRNUC	1H
CTEMP	26.8 c
SLVNT	CDCL3
EXREF	7.26 ppm
BF	0.12 Hz
RGAIN	12

C:\Users\Takamura\Documents\"Še˜•¶\2013\SBD C79-C97 fragment\BJOC\ì¬'—Þ\2013 04 12\NMR\alice file\TF-531 13CNMR data.als

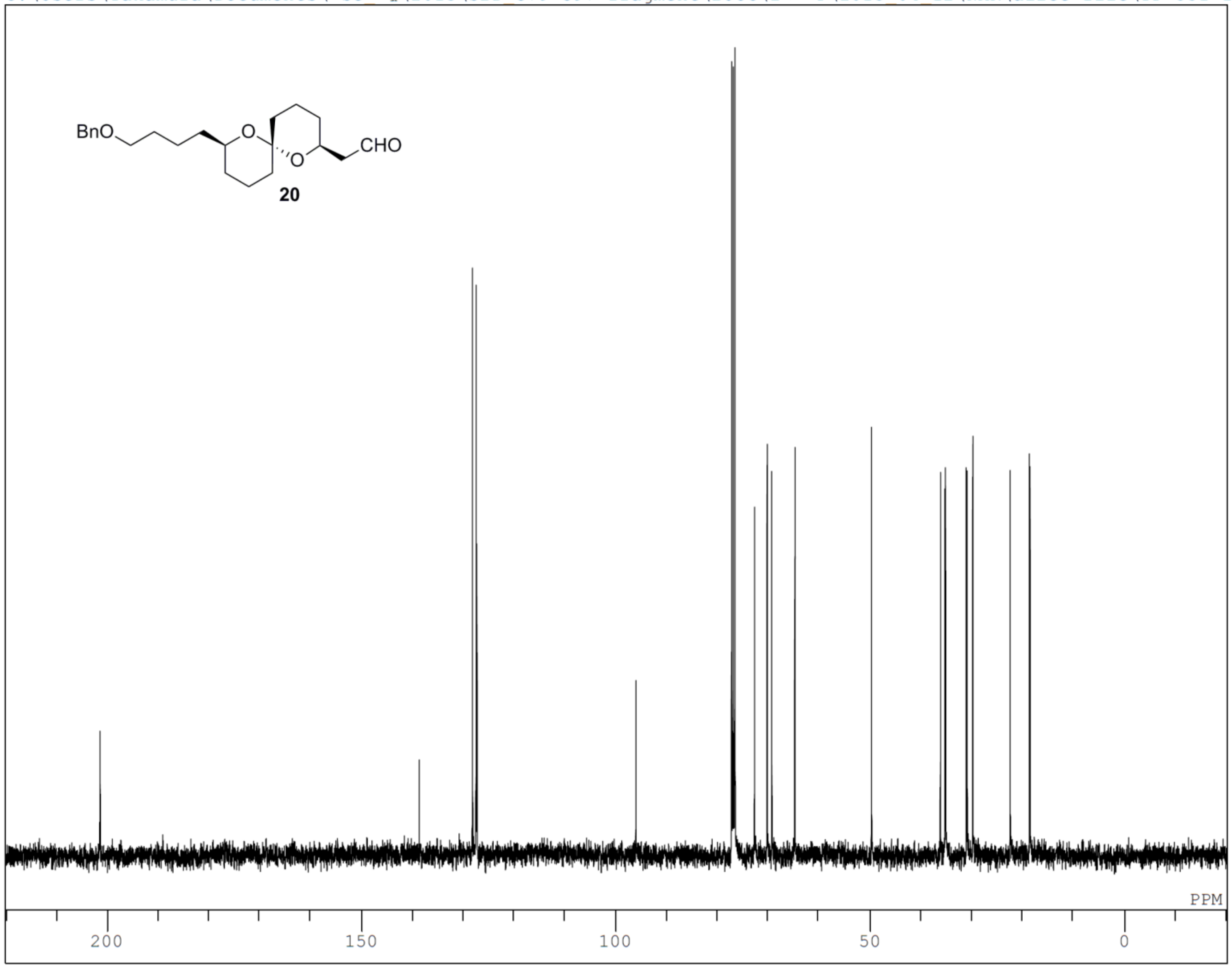

DFILE TF-531 13CNMR data.
COMNT TF-531 13CNMR
DATIM Mon Sep 10 13:52:20
OBNUC 13C
EXMOD BCM
OBFRQ 100.40 MHz
OBSET 125.00 KHz
OBFIN 10500.00 Hz
POINT 32768
FREQU 27173.90 Hz
SCANS 358
ACQTM 1.2059 sec
PD 1.7940 sec
PW1 6.80 usec
IRNUC 1H
CTEMP 30.6 c
SLVNT CDCL3
EXREF 77.00 ppm
BF 2.00 Hz
RGAIN 23

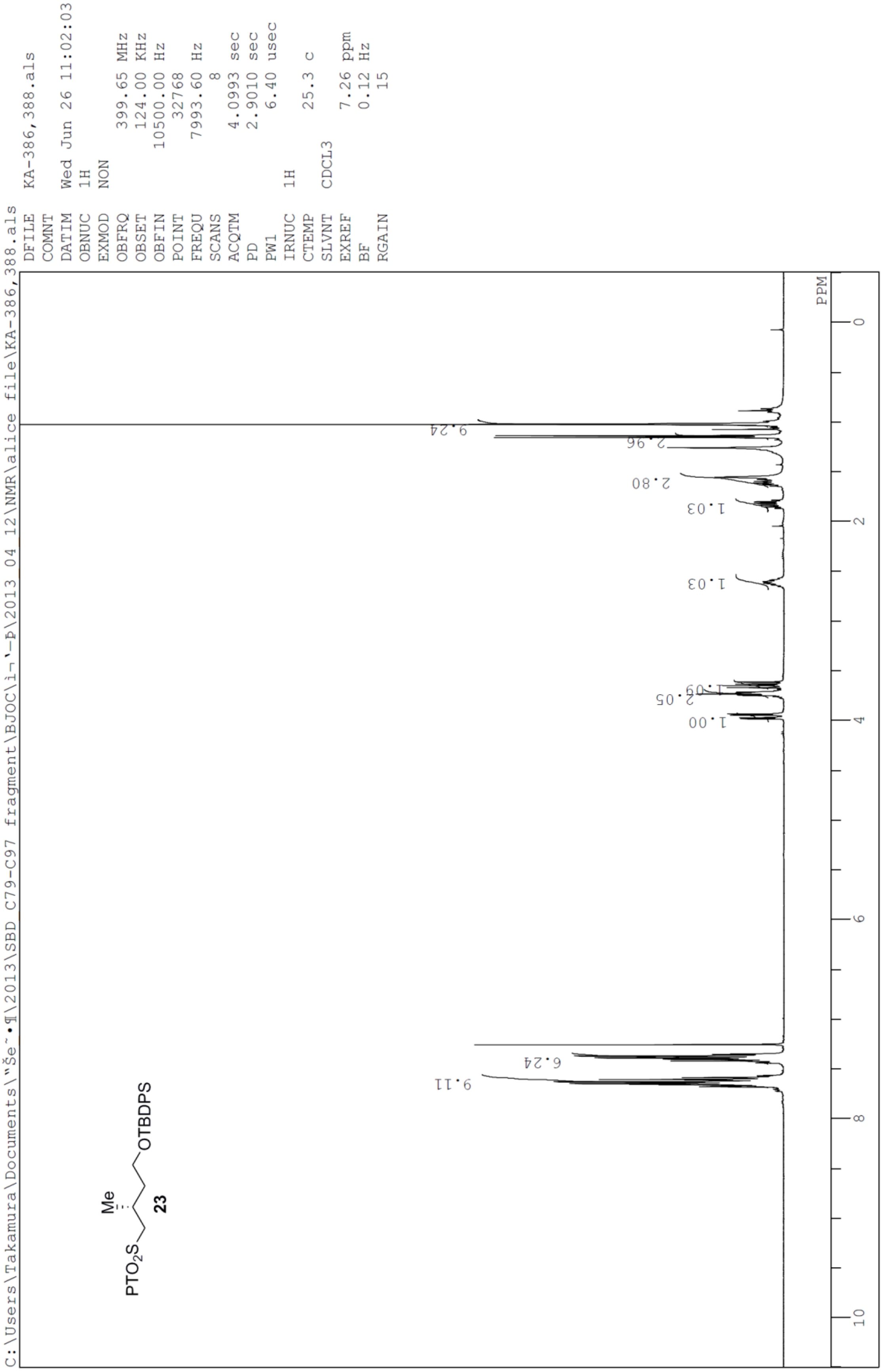

C:\Users\Takamura\Documents\"Še ˜ •¶\2013\SBD C79-C97 fragment\BJOC\i¬'-Þ\2013 04 12\NMR\alice file\KA-386,388.als
DFILE KA-386,388.als
COMNT
DATIM Wed Jun 26 11:02:03
OBNUC 1H
EXMOD NON
OBFRQ 399.65 MHz
OBSET 124.00 KHz
OBFIN 10500.00 Hz
POINT 32768
FREQU 7993.60 Hz
SCANS 8
ACQTM 4.0993 sec
PD 2.9010 sec
PW1 6.40 usec
IRNUC 1H
CTEMP 25.3 c
SLVNT CDCL3
EXREF 7.26 ppm
BF 0.12 Hz
RGAIN 15
PTO2S
Me
OTBDPS
23
9.11
6.24
1.00
2.05
1.09
1.03
1.03
2.80
2.96
9.24
PPM
0
2
4
6
8
10

C:\Users\Takamura\Documents\"Še˜•¶\2013\SBD C79-C97 fragment\BJOC\ì¬'—Þ\2013 04 12\NMR\alice file\KA-386,388 13C.als

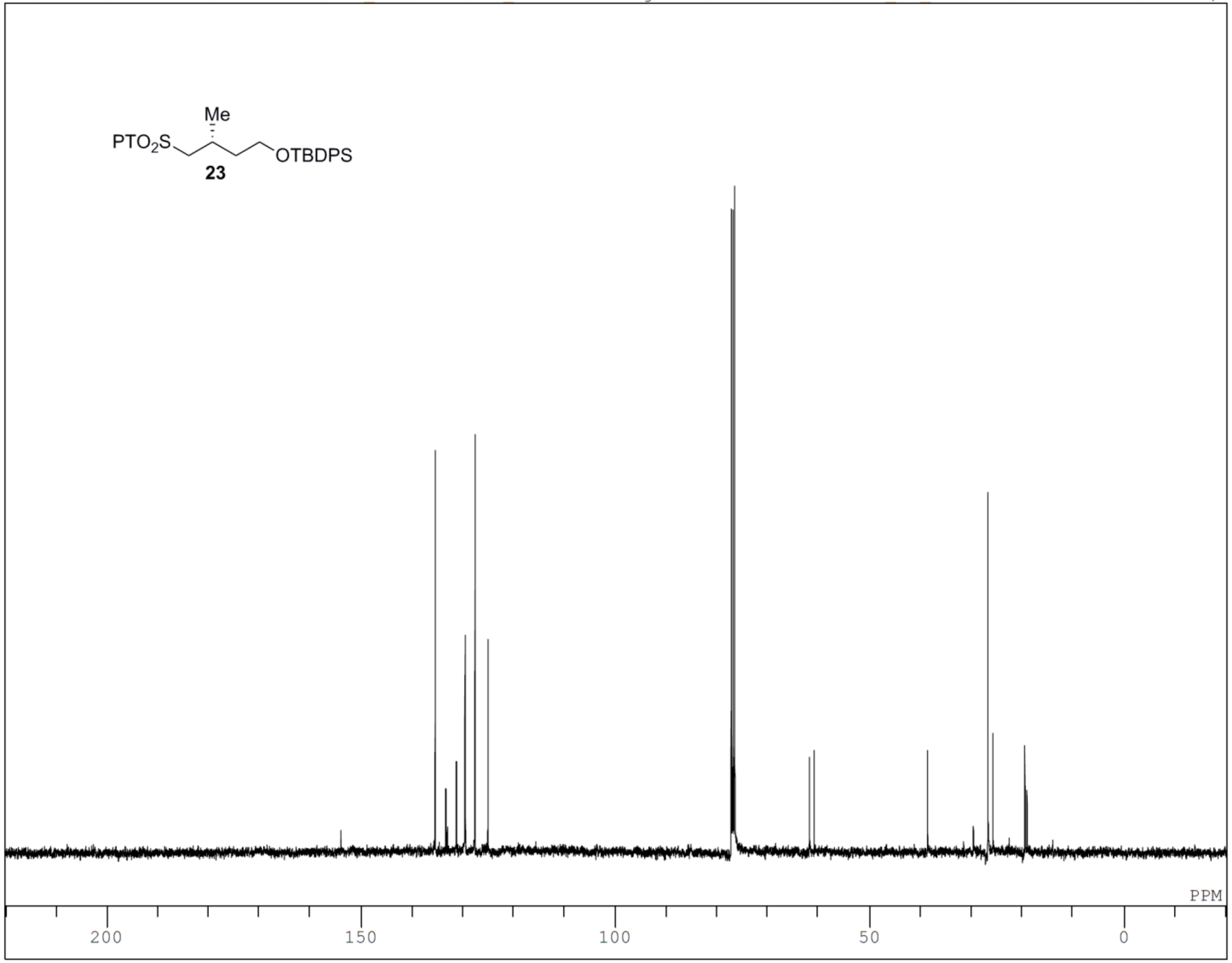

DFILE	KA-386,388 13C.als
COMNT	
DATIM	Wed Jun 26 12:06:38
OBNUC	13C
EXMOD	BCM
OBFRQ	100.40 MHz
OBSET	125.00 KHz
OBFIN	10500.00 Hz
POINT	32768
FREQU	27173.90 Hz
SCANS	1210
ACQTM	1.2059 sec
PD	1.7940 sec
PW1	6.80 usec
IRNUC	1H
CTEMP	29.2 c
SLVNT	CDCL3
EXREF	77.00 ppm
BF	2.00 Hz
RGAIN	23

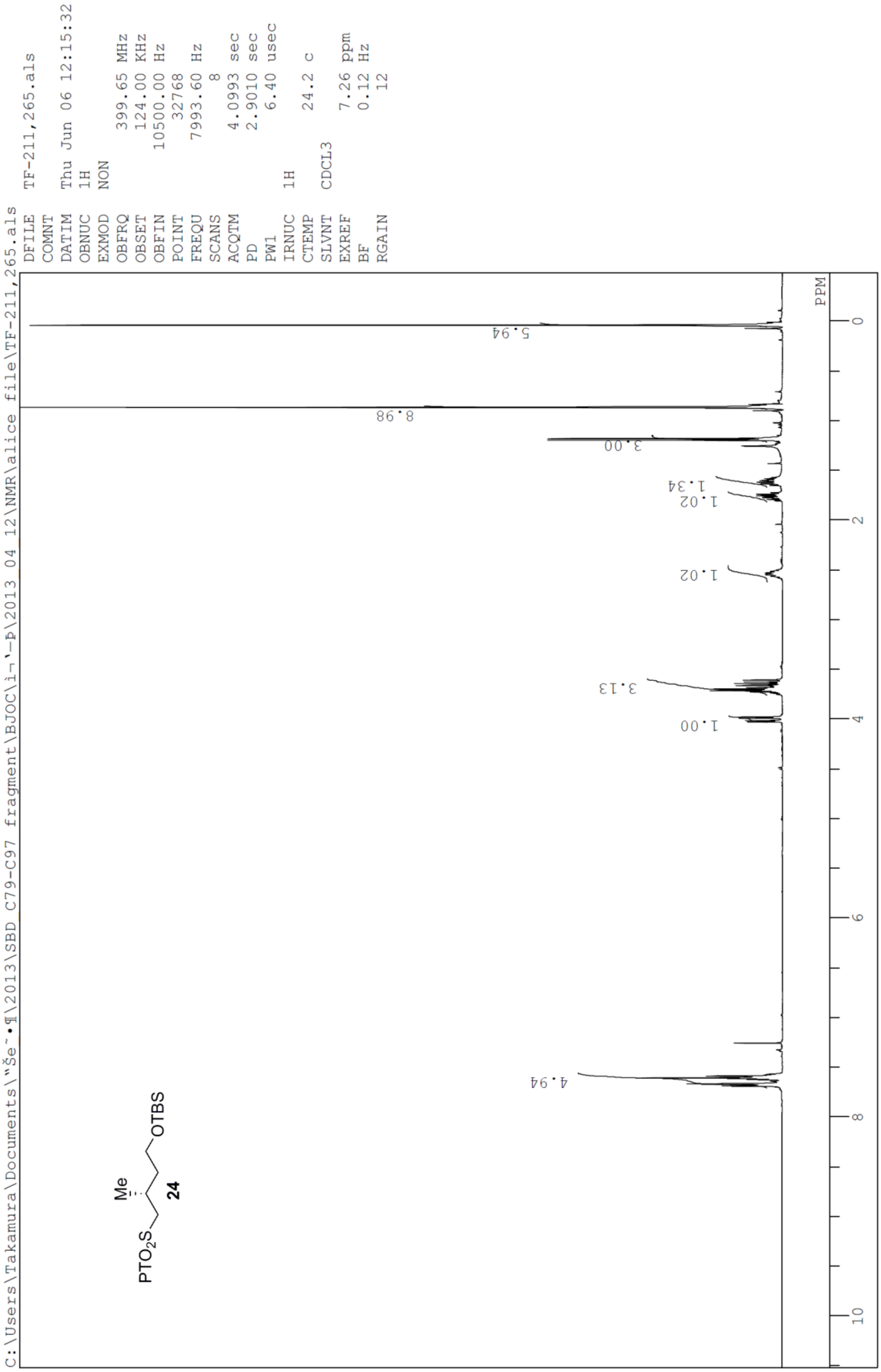
C:\Users\Takamura\Documents\"Še¨•¶\2013\SBD C79-C97 fragment\BJOC\i¬'-Þ\2013 04 12\NMR\alice file\TF-211,265.als
DFILE TF-211,265.als
COMNT
DATIM Thu Jun 06 12:15:32
OBNUC 1H
EXMOD NON
OBFRQ 399.65 MHz
OBSET 124.00 KHz
OBFIN 10500.00 Hz
POINT 32768
FREQU 7993.60 Hz
SCANS 8
ACQTM 4.0993 sec
PD 2.9010 sec
PW1 6.40 usec
IRNUC 1H
CTEMP 24.2 c
SLVNT CDCL3
EXREF 7.26 ppm
BF 0.12 Hz
RGAIN 12
PTO2S
Me
OTBS
24
PPM
5.94
8.98
3.00
1.34
1.02
1.02
3.13
1.00
4.94
0
2
4
6
8
10

C:\Users\Takamura\Documents\"Še˜•¶\2013\SBD C79-C97 fragment\BJOC\ì¬'–Þ\2013 04 12\NMR\alice file\TF-211,265@13C.als

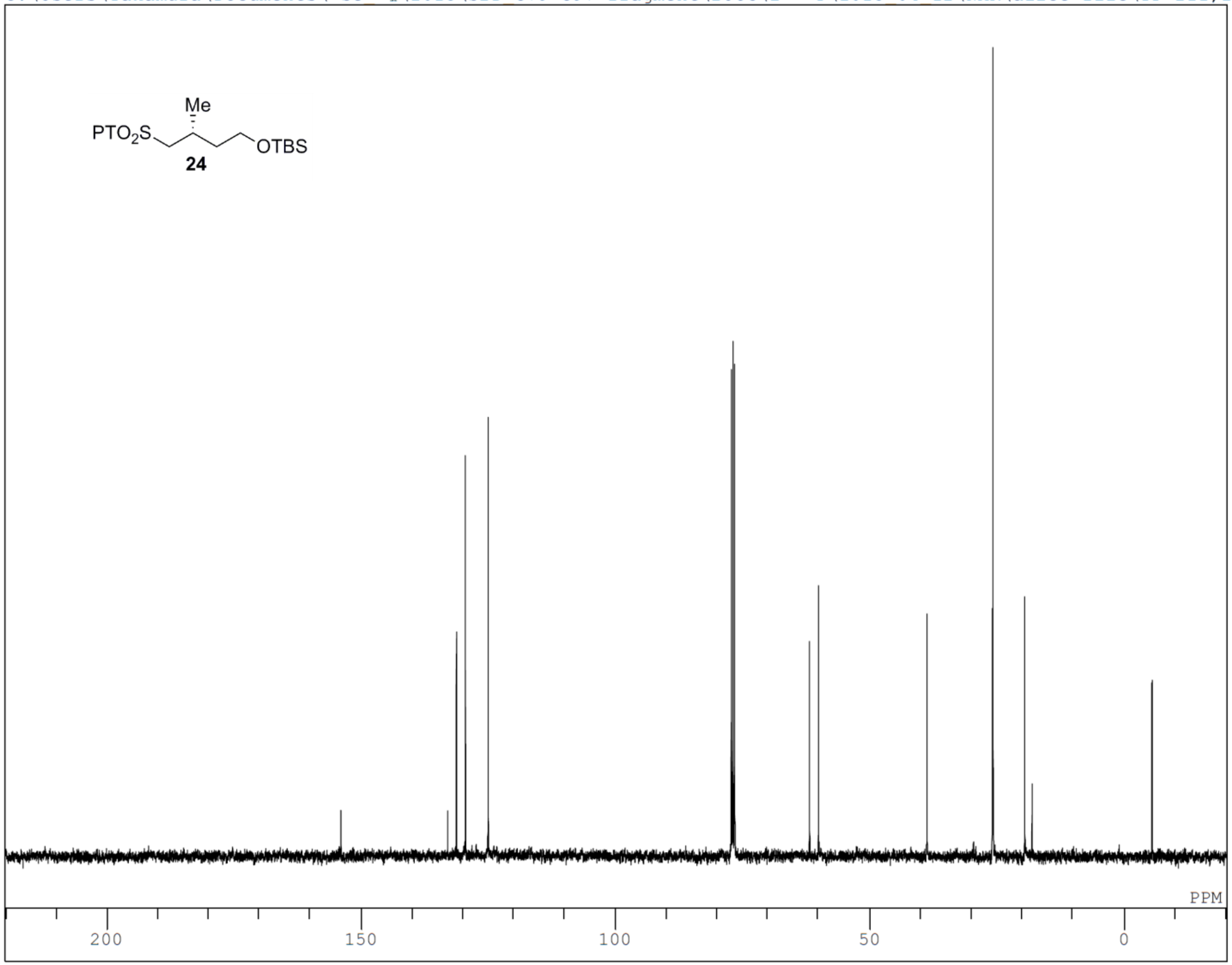


```
DFILE   TF-211,265@13C.als
COMNT
DATIM   Wed Jun 05 12:29:43
OBNUC   13C
EXMOD   BCM
OBFRQ        100.40 MHz
OBSET        125.00 KHz
OBFIN      10500.00 Hz
POINT         32768
FREQU      27173.90 Hz
SCANS           302
ACQTM        1.2059 sec
PD           1.7940 sec
PW1            6.80 usec
IRNUC   1H
CTEMP          26.9 c
SLVNT   CDCL3
EXREF         77.00 ppm
BF             2.00 Hz
RGAIN            23
```

C:\Users\Takamura\Documents\"Še˜•¶\2013\SBD C79-C97 fragment\BJOC\ì¬'–Þ\2013 04 12\NMR\alice file\KA-382,383.als

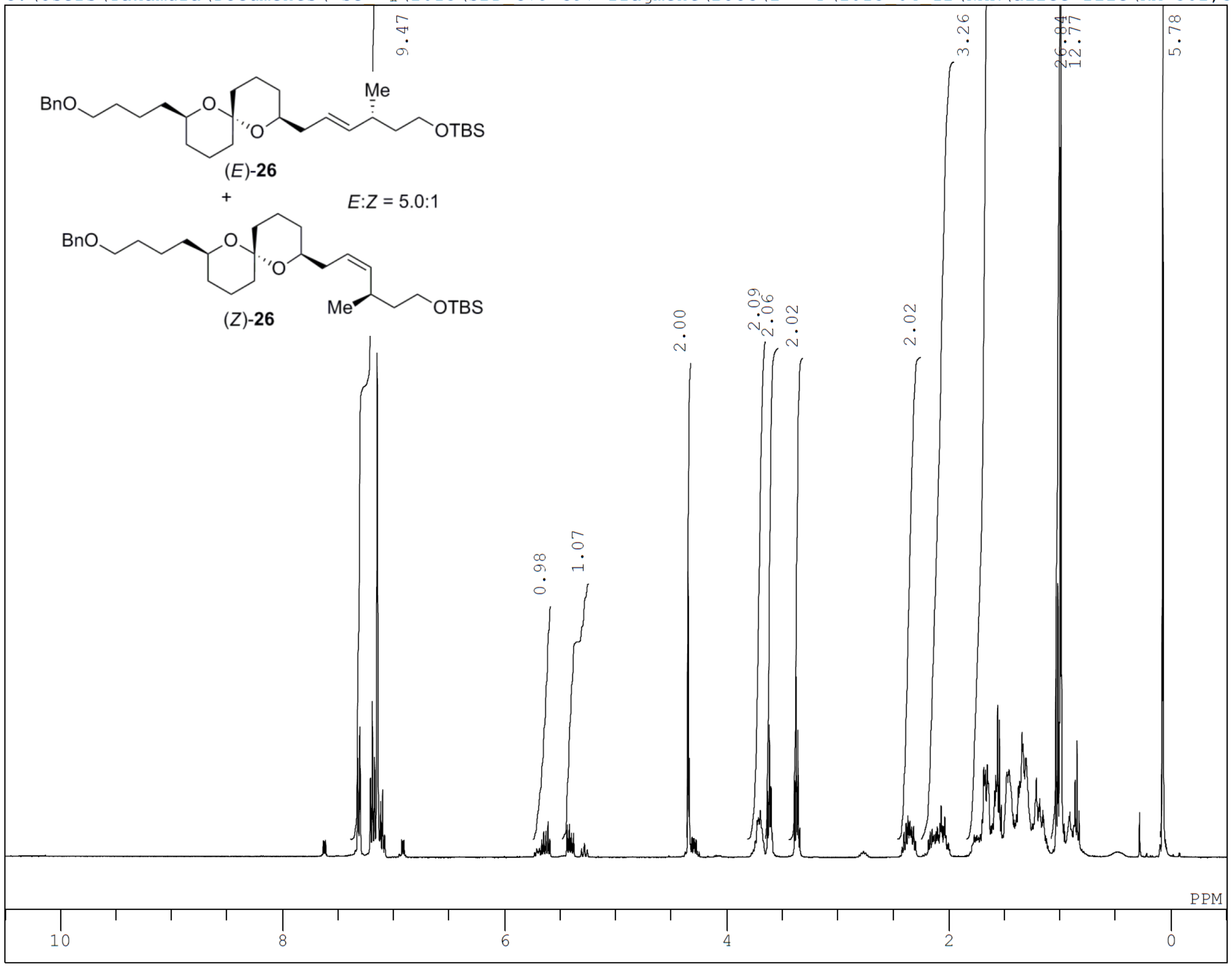

DFILE	KA-382,383.als
COMNT	
DATIM	Wed Jun 12 16:04:43
OBNUC	1H
EXMOD	NON
OBFRQ	399.65 MHz
OBSET	124.00 KHz
OBFIN	10500.00 Hz
POINT	32768
FREQU	7993.60 Hz
SCANS	8
ACQTM	4.0993 sec
PD	2.9010 sec
PW1	6.40 usec
IRNUC	1H
CTEMP	24.0 c
SLVNT	C6D6
EXREF	7.15 ppm
BF	0.12 Hz
RGAIN	13

C:\Users\Takamura\Documents\"Še˜•¶\2013\SBD C79-C97 fragment\BJOC\ì¬'—Þ\2013 04 12\NMR\alice file\KA-382,383 13C.als

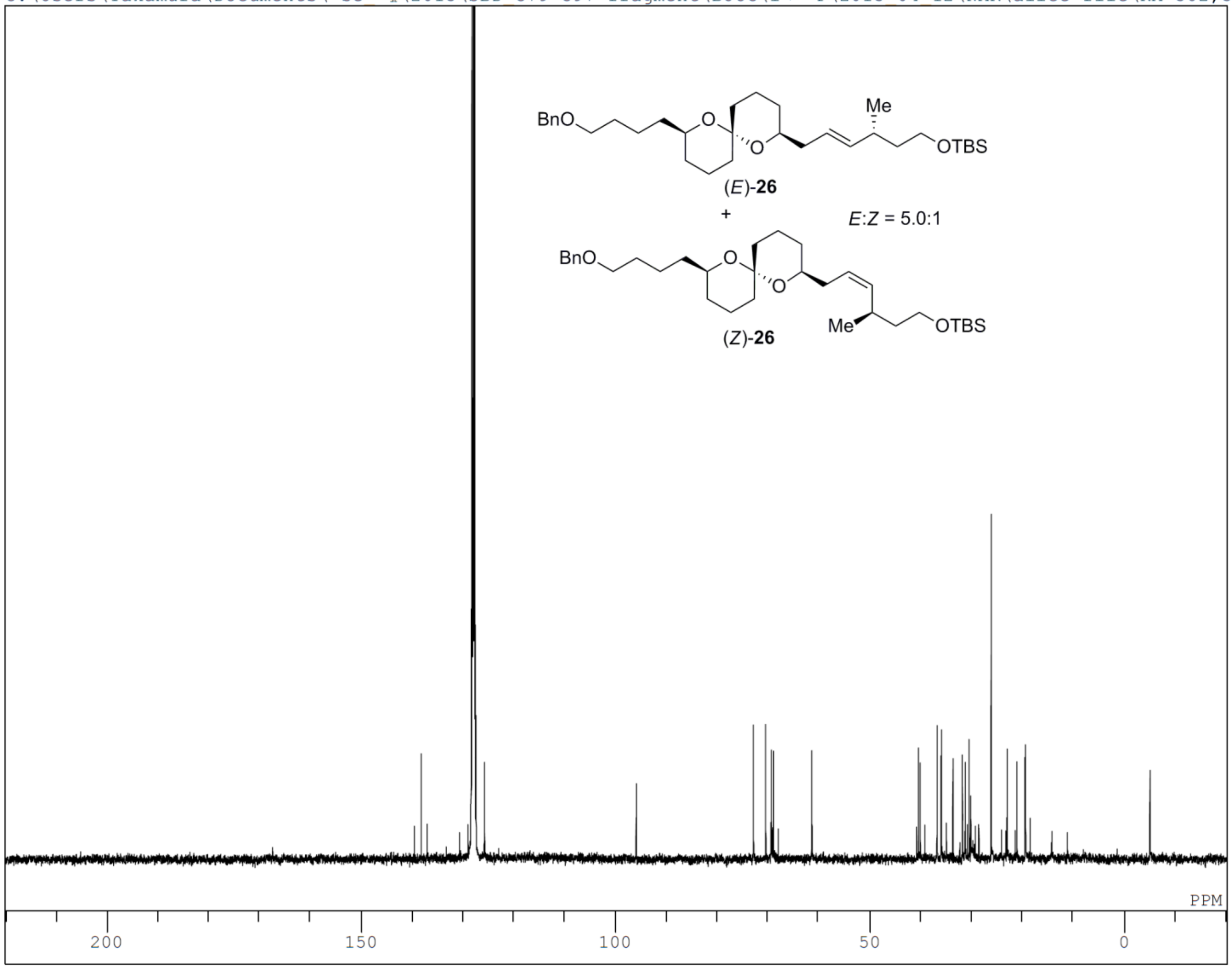

DFILE	KA-382,383 13C.als
COMNT	
DATIM	Wed Jun 12 16:59:00
OBNUC	13C
EXMOD	BCM
OBFRQ	100.40 MHz
OBSET	125.00 KHz
OBFIN	10500.00 Hz
POINT	32768
FREQU	27173.90 Hz
SCANS	903
ACQTM	1.2059 sec
PD	1.7940 sec
PW1	6.80 usec
IRNUC	1H
CTEMP	27.7 c
SLVNT	C6D6
EXREF	128.00 ppm
BF	2.00 Hz
RGAIN	18

C:\Users\Takamura\Documents\"Še˜•¶\2013\SBD C79-C97 fragment\BJOC\ì¬'—Þ\2013 04 12\NMR\alice file\TF-179-3 date.als

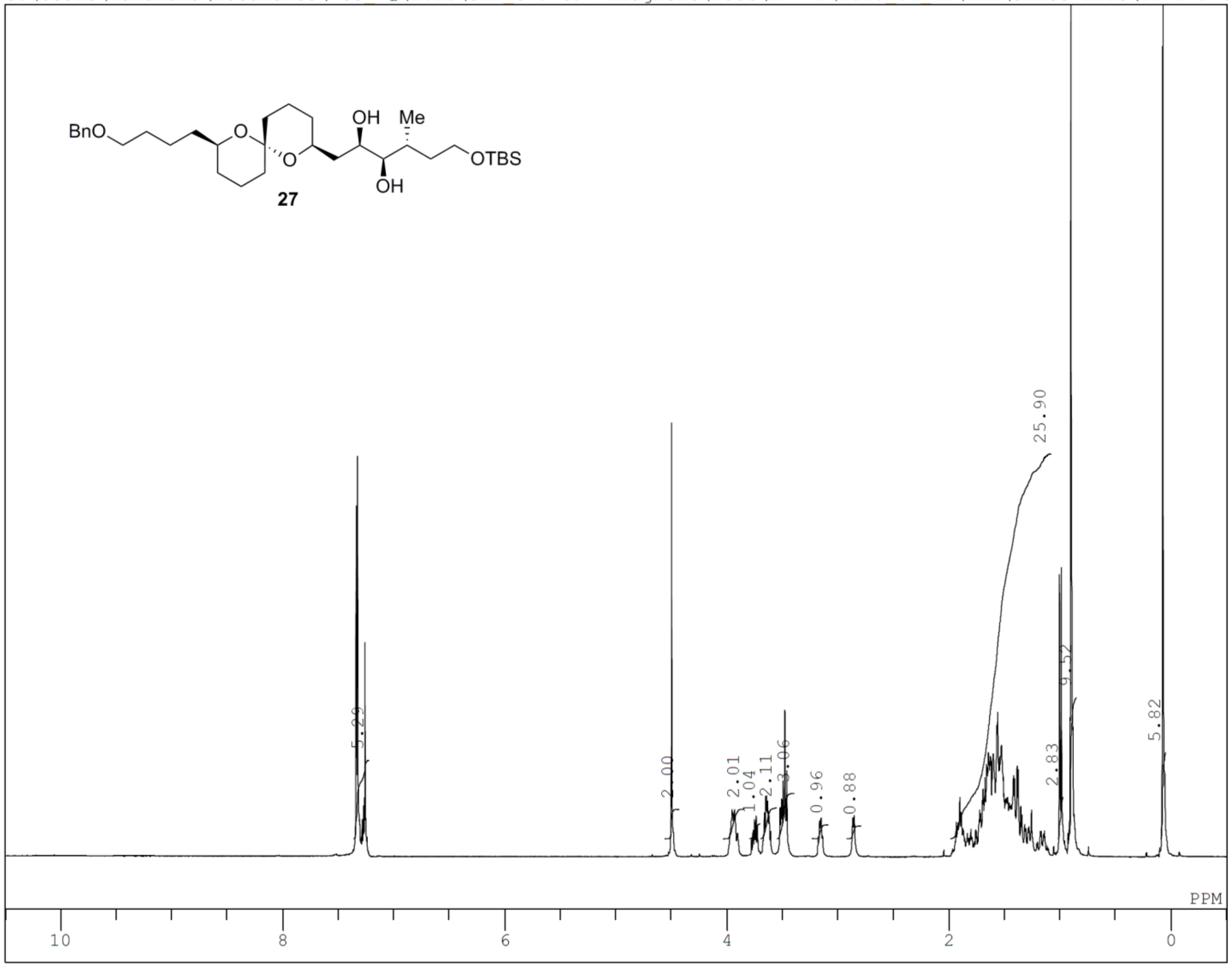

DFILE	TF-179-3 date.als
COMNT	TF-179-3
DATIM	Fri Mar 18 16:56:07
OBNUC	1H
EXMOD	NON
OBFRQ	399.65 MHz
OBSET	124.00 KHz
OBFIN	10500.00 Hz
POINT	32768
FREQU	7993.60 Hz
SCANS	8
ACQTM	4.0993 sec
PD	2.9010 sec
PW1	6.40 usec
IRNUC	1H
CTEMP	22.9 c
SLVNT	CDCL3
EXREF	7.26 ppm
BF	0.12 Hz
RGAIN	13

C:\Users\Takamura\Documents\“Še˜•¶\2013\SBD C79-C97 fragment\BJOC\ì¬’—Þ\2013 04 12\NMR\alice file\TF-179-3 13C.als

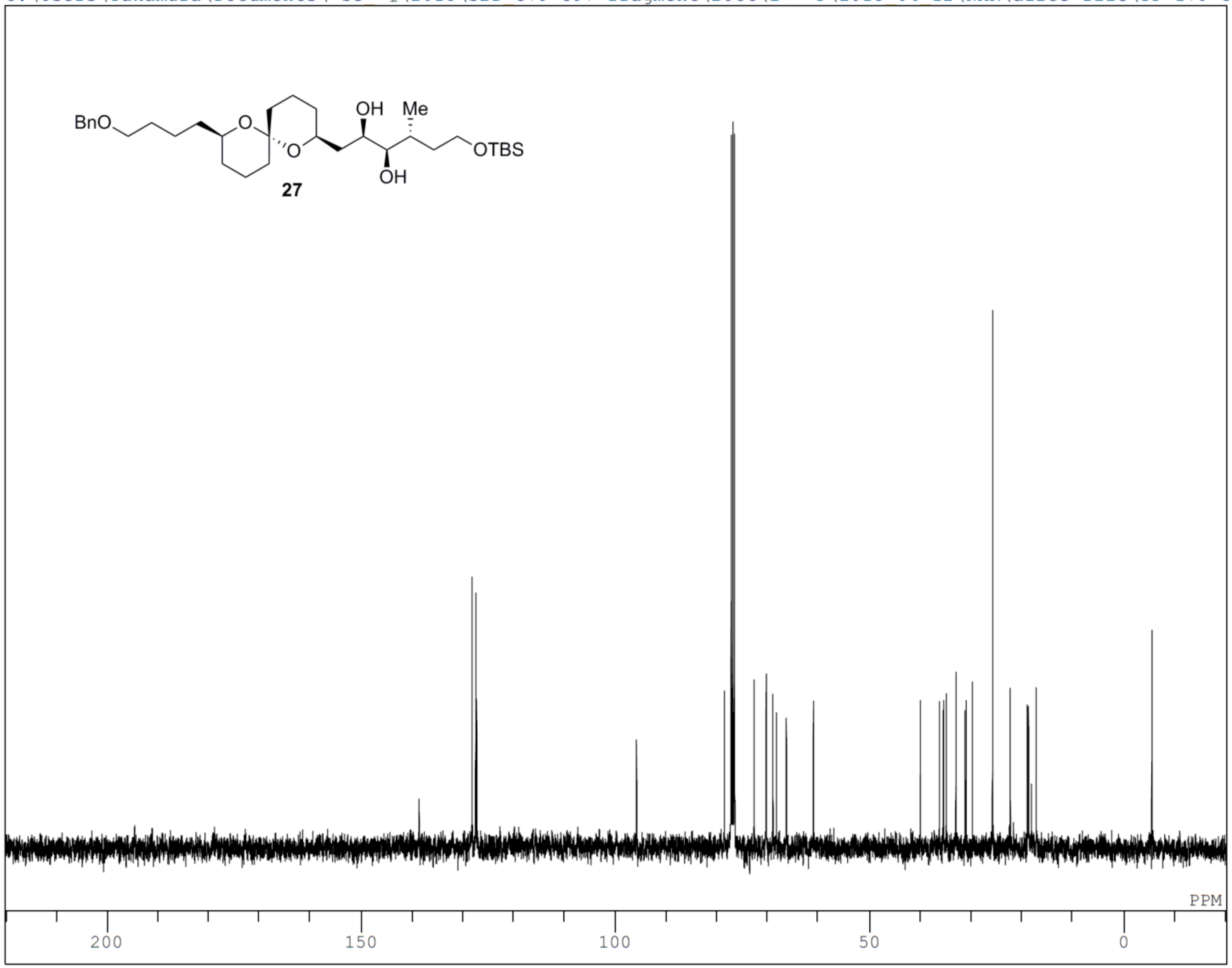

DFILE	TF-179-3 13C.als
COMNT	TF-179-3-13C
DATIM	Fri Mar 18 17:10:31
OBNUC	13C
EXMOD	BCM
OBFRQ	100.40 MHz
OBSET	125.00 KHz
OBFIN	10500.00 Hz
POINT	32768
FREQU	27173.90 Hz
SCANS	158
ACQTM	1.2059 sec
PD	1.7940 sec
PW1	6.80 usec
IRNUC	1H
CTEMP	25.1 c
SLVNT	CDCL3
EXREF	77.00 ppm
BF	2.00 Hz
RGAIN	24

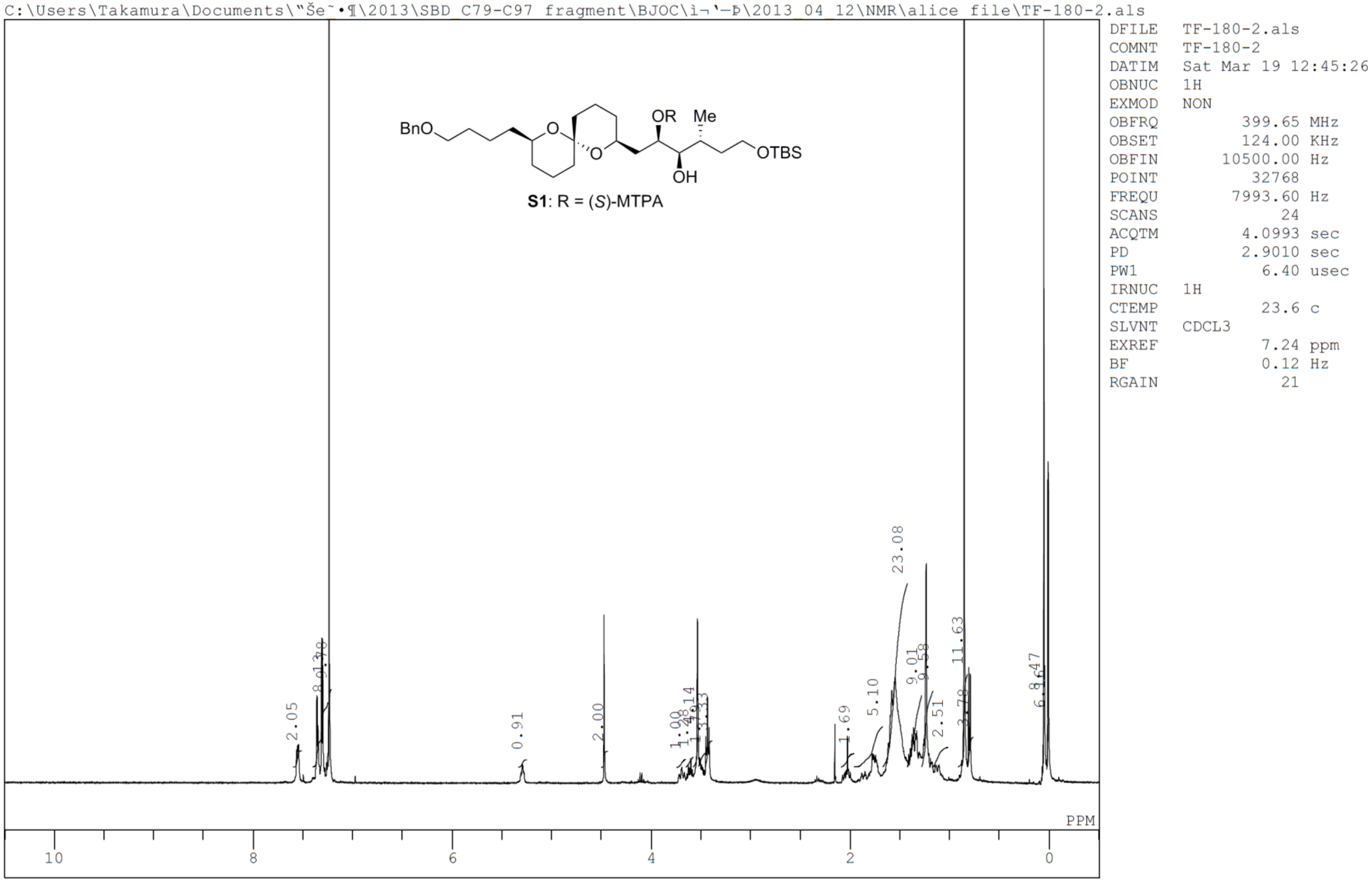
C:\Users\Takamura\Documents\"Še˜•¶\2013\SBD C79-C97 fragment\BJOC\ì¬'—Þ\2013 04 12\NMR\alice file\TF-180-2.als
DFILE TF-180-2.als
COMNT TF-180-2
DATIM Sat Mar 19 12:45:26
OBNUC 1H
EXMOD NON
OBFRQ 399.65 MHz
OBSET 124.00 KHz
OBFIN 10500.00 Hz
POINT 32768
FREQU 7993.60 Hz
SCANS 24
ACQTM 4.0993 sec
PD 2.9010 sec
PW1 6.40 usec
IRNUC 1H
CTEMP 23.6 c
SLVNT CDCL3
EXREF 7.24 ppm
BF 0.12 Hz
RGAIN 21
BnO
O
O
OR
Me
OH
OTBS
S1: R = (S)-MTPA
2.05
0.91
2.00
1.69
5.10
23.08
9.01
9.58
2.51
11.63
PPM
10
8
6
4
2
0

C:\Users\Takamura\Documents\"Še˜•¶\2013\SBD C79-C97 fragment\BJOC\ì¬'—Þ\2013 04 12\NMR\alice file\TF-177-2.als

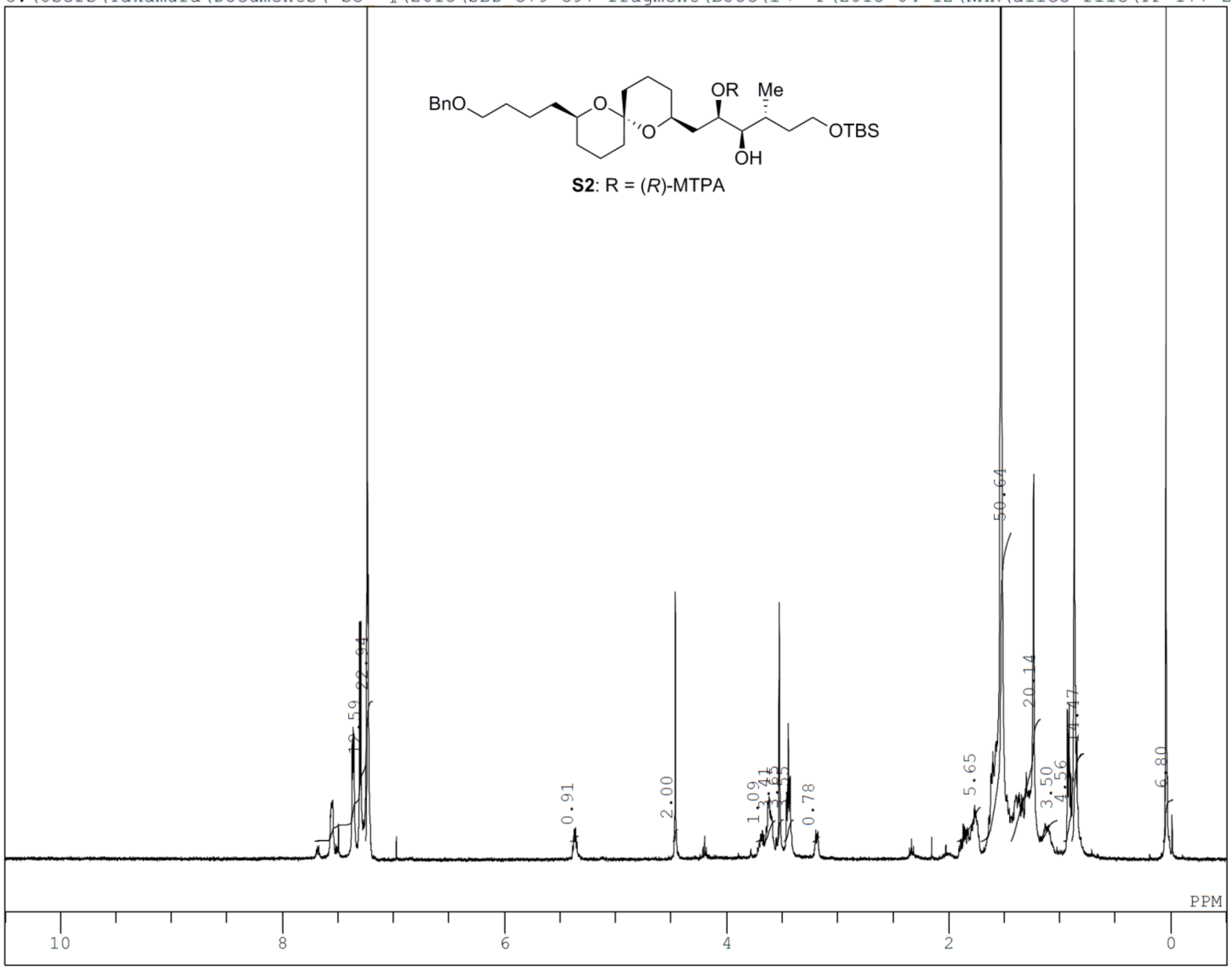

DFILE	TF-177-2.als
COMNT	TF-177-2
DATIM	Mon Mar 14 19:50:02
OBNUC	1H
EXMOD	NON
OBFRQ	399.65 MHz
OBSET	124.00 KHz
OBFIN	10500.00 Hz
POINT	32768
FREQU	7993.60 Hz
SCANS	80
ACQTM	4.0993 sec
PD	2.9010 sec
PW1	6.40 usec
IRNUC	1H
CTEMP	23.7 c
SLVNT	CDCL3
EXREF	7.24 ppm
BF	0.12 Hz
RGAIN	22

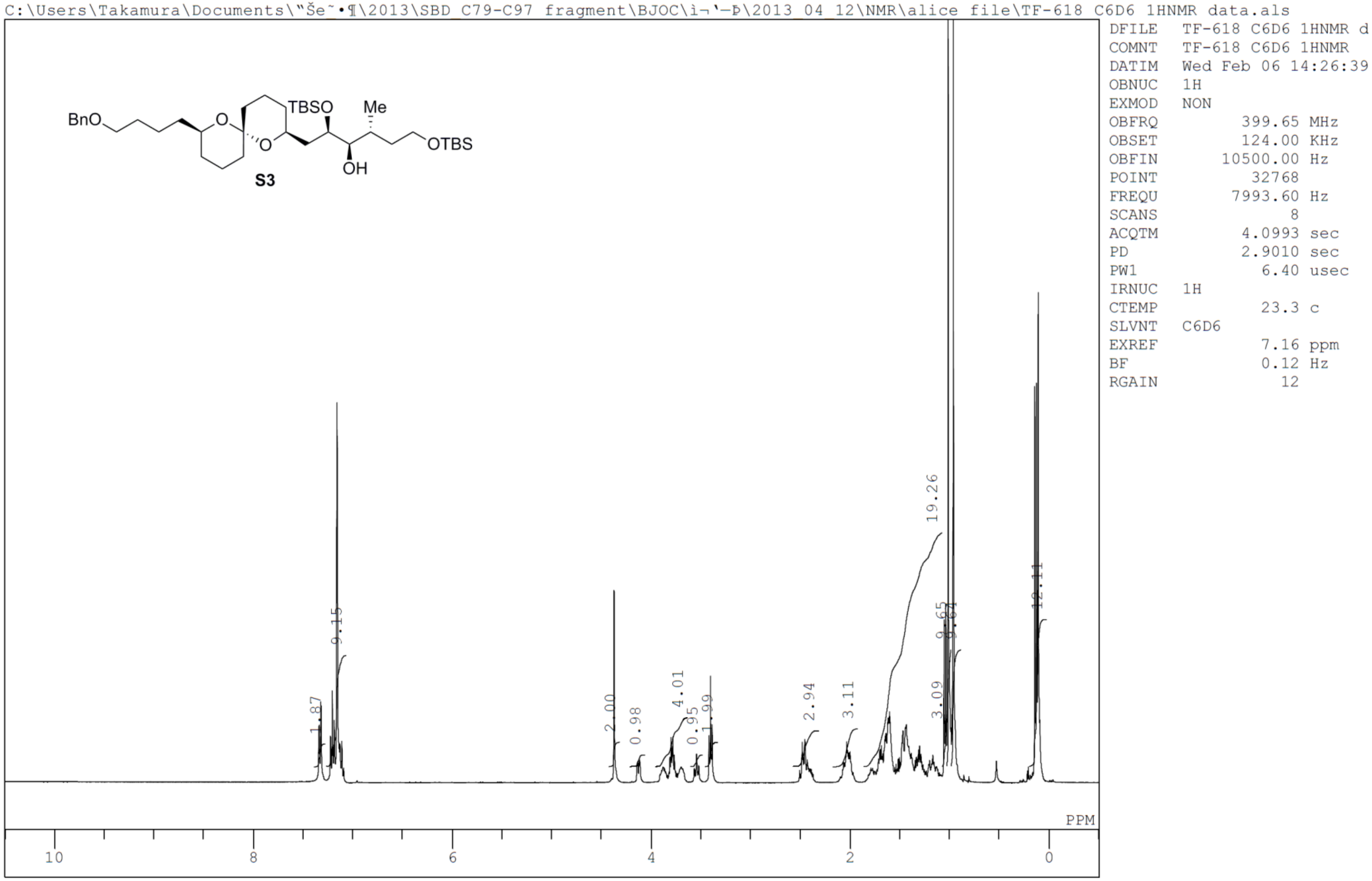
C:\Users\Takamura\Documents\"Še˜•¶\2013\SBD C79-C97 fragment\BJOC\ì¬'—Þ\2013 04 12\NMR\alice file\TF-618 C6D6 1HNMR data.als
DFILE TF-618 C6D6 1HNMR d
COMNT TF-618 C6D6 1HNMR
DATIM Wed Feb 06 14:26:39
OBNUC 1H
EXMOD NON
OBFRQ 399.65 MHz
OBSET 124.00 KHz
OBFIN 10500.00 Hz
POINT 32768
FREQU 7993.60 Hz
SCANS 8
ACQTM 4.0993 sec
PD 2.9010 sec
PW1 6.40 usec
IRNUC 1H
CTEMP 23.3 c
SLVNT C6D6
EXREF 7.16 ppm
BF 0.12 Hz
RGAIN 12
BnO
TBSO
Me
OTBS
OH
S3
1.87
9.15
2.00
0.98
4.01
0.95
1.99
2.94
3.11
3.09
19.26
9.65
12.11
PPM
10
8
6
4
2
0

C:\Users\Takamura\Documents\“Še˜•¶\2013\SBD C79-C97 fragment\BJOC\ì¬'–Þ\2013 04 12\NMR\alice file\TF-618 C6D6 13CNMR data.als

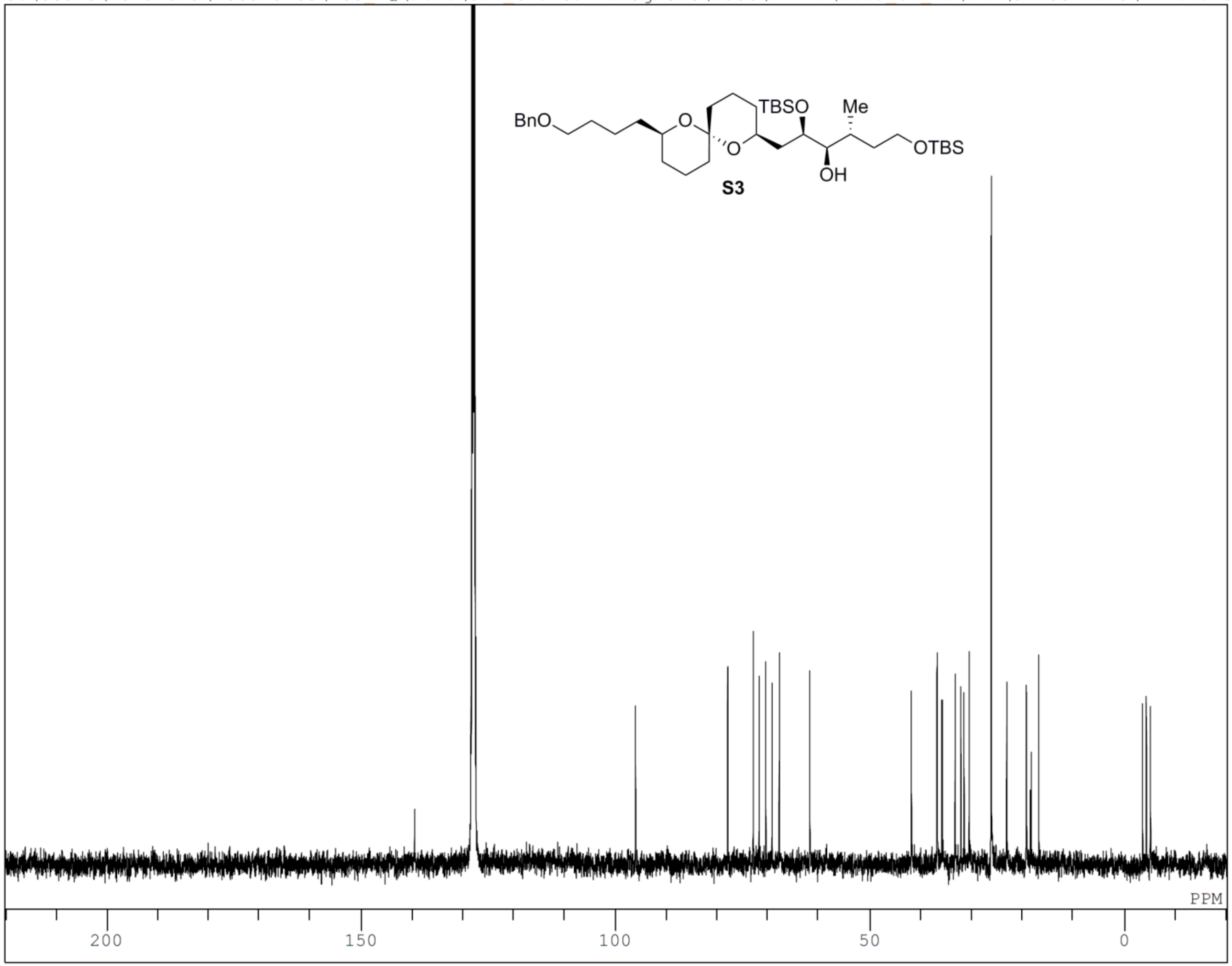

DFILE	TF-618 C6D6 13CNMR
COMNT	TF-618 C6D6 13CNMR
DATIM	Wed Feb 06 14:54:54
OBNUC	13C
EXMOD	BCM
OBFRQ	100.40 MHz
OBSET	125.00 KHz
OBFIN	10500.00 Hz
POINT	32768
FREQU	27173.90 Hz
SCANS	436
ACQTM	1.2059 sec
PD	1.7940 sec
PW1	6.80 usec
IRNUC	1H
CTEMP	26.4 c
SLVNT	C6D6
EXREF	128.00 ppm
BF	2.00 Hz
RGAIN	18

C:\Users\Takamura\Documents\"Še˜•¶\2013\SBD C79-C97 fragment\BJOC\ì¬'–Þ\2013 04 12\NMR\alice file\TF-619+222 (S)-ester 1HNMR data.al

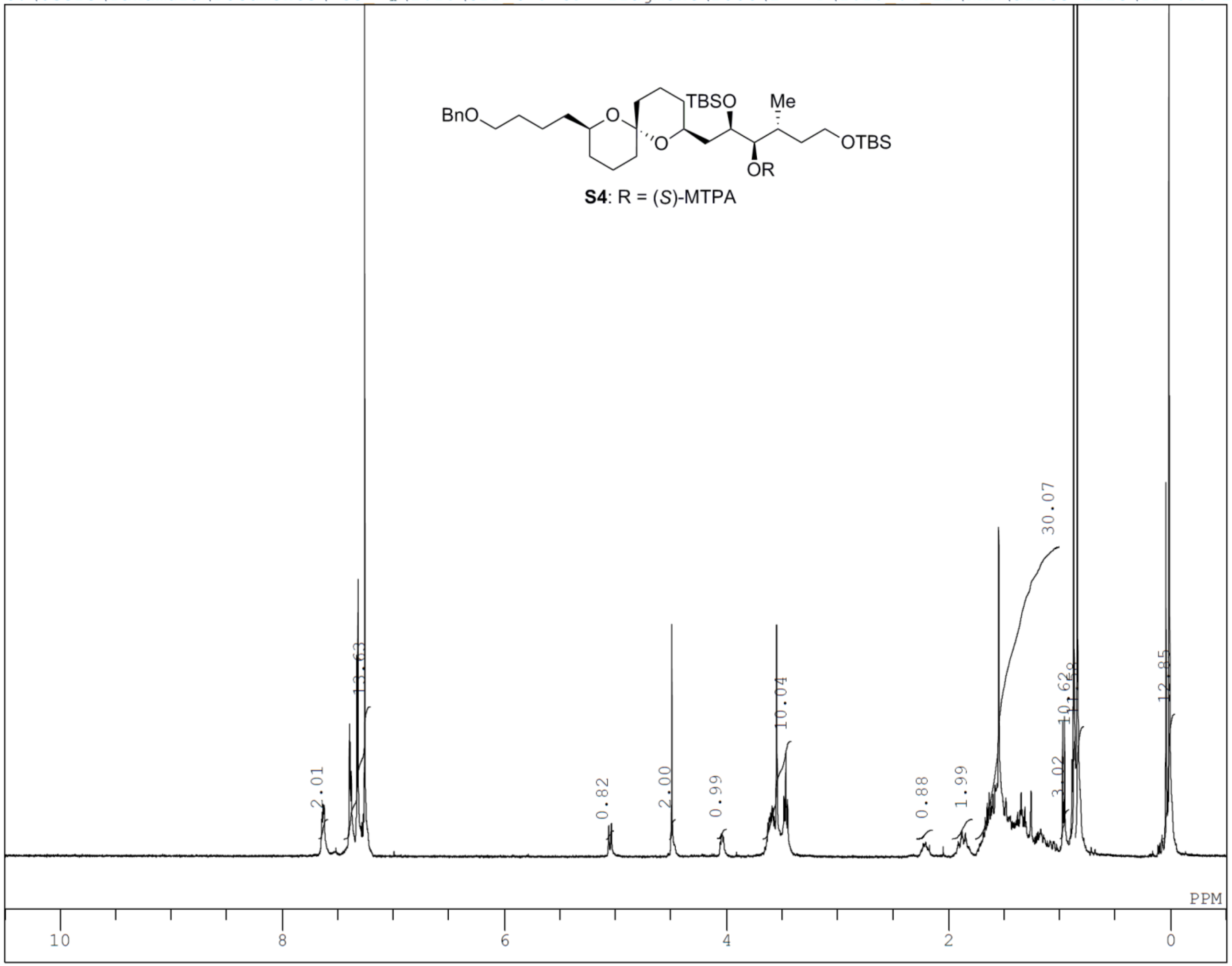


```
DFILE   TF-619+222 (S)-este
COMNT   TF-619,222 1HNMR
DATIM   Thu Feb 07 19:41:18
OBNUC   1H
EXMOD   NON
OBFRQ        399.65 MHz
OBSET        124.00 KHz
OBFIN      10500.00 Hz
POINT         32768
FREQU       7993.60 Hz
SCANS             8
ACQTM        4.0993 sec
PD           2.9010 sec
PW1            6.40 usec
IRNUC   1H
CTEMP          23.3 c
SLVNT   CDCL3
EXREF          7.26 ppm
BF             0.12 Hz
RGAIN            18
```

C:\Users\Takamura\Documents\"Še˜•¶\2013\SBD C79-C97 fragment\BJOC\ì¬'—Þ\2013 04 12\NMR\alice file\TF-619+222 (S)-ester 13CNMR data.a

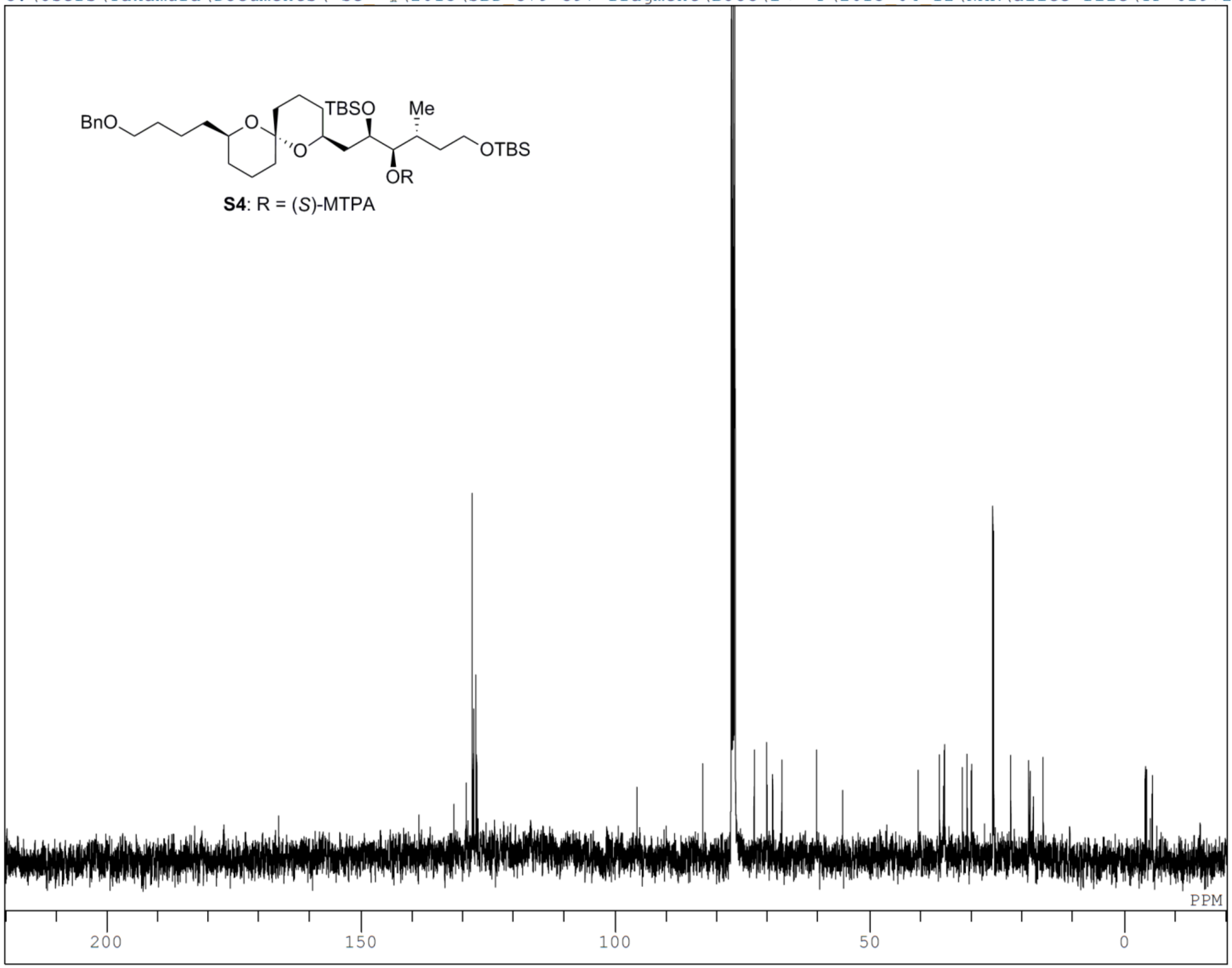

DFILE	TF-619+222 (S)-este
COMNT	TF-619,222 13CNMR
DATIM	Thu Feb 07 21:51:08
OBNUC	13C
EXMOD	BCM
OBFRQ	100.40 MHz
OBSET	125.00 KHz
OBFIN	10500.00 Hz
POINT	32768
FREQU	27173.90 Hz
SCANS	1874
ACQTM	1.2059 sec
PD	1.7940 sec
PW1	6.80 usec
IRNUC	1H
CTEMP	25.9 c
SLVNT	CDCL3
EXREF	77.00 ppm
BF	2.00 Hz
RGAIN	24

C:\Users\Takamura\Documents\"Še˜•¶\2013\SBD C79-C97 fragment\BJOC\ì¬'—Þ\2013 04 12\NMR\alice file\TF-620+221 (R)-ester 1HNMR data.al

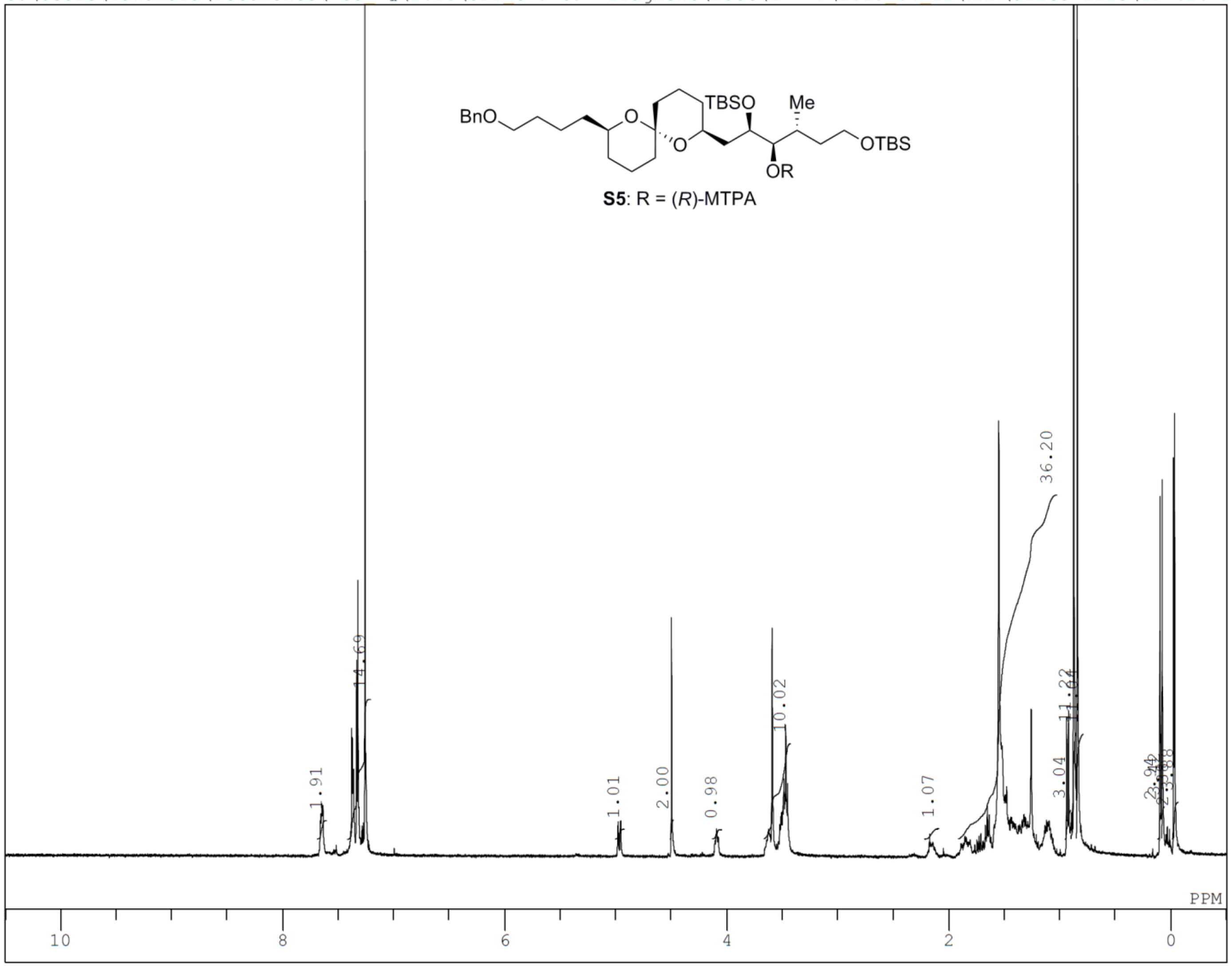

DFILE	TF-620+221 (R)-este
COMNT	TF-620,221 1HNMR
DATIM	Thu Feb 07 23:29:18
OBNUC	1H
EXMOD	NON
OBFRQ	399.65 MHz
OBSET	124.00 KHz
OBFIN	10500.00 Hz
POINT	32768
FREQU	7993.60 Hz
SCANS	8
ACQTM	4.0993 sec
PD	2.9010 sec
PW1	6.40 usec
IRNUC	1H
CTEMP	23.1 c
SLVNT	CDCL3
EXREF	7.26 ppm
BF	0.12 Hz
RGAIN	20

C:\Users\Takamura\Documents\“Še˜•¶\2013\SBD_C79-C97 fragment\BJOC\ì¬'—Þ\2013_04_12\NMR\alice file\TF-620+221 (R)-ester 13CNMR data.a

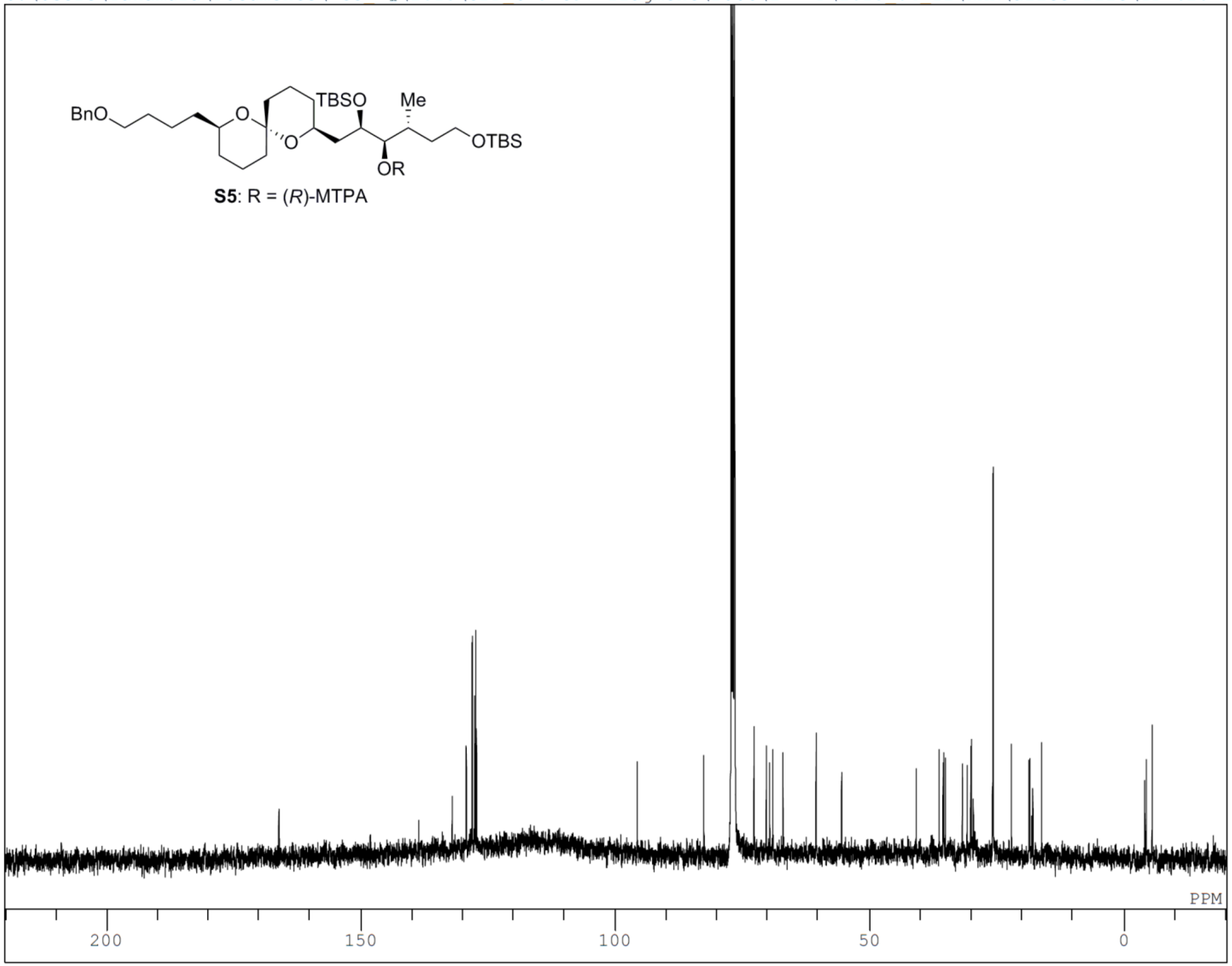

DFILE	TF-620+221 (R)-este
COMNT	TF-620,221 13CNMR
DATIM	Fri Feb 08 09:11:08
OBNUC	13C
EXMOD	BCM
OBFRQ	100.40 MHz
OBSET	125.00 KHz
OBFIN	10500.00 Hz
POINT	32768
FREQU	27173.90 Hz
SCANS	10922
ACQTM	1.2059 sec
PD	1.7940 sec
PW1	6.80 usec
IRNUC	1H
CTEMP	25.2 c
SLVNT	CDCL3
EXREF	77.00 ppm
BF	2.00 Hz
RGAIN	24
